# Targeting the Correa’s cascade of gastric cancer: the potential role of phytochemicals and autophagy modulation

**DOI:** 10.3389/fphar.2026.1689279

**Published:** 2026-09-25

**Authors:** Tingyao Wang, Fuming Yao, Ruilin Li, Jijun Zheng, Hebin Zhang, Jia Ma, Yueqiang Wen, Xiao Ma, Jinhao Zeng

**Affiliations:** 1 TOM Prevention and Treatment of Metabolic and Chronic Diseases Key Laboratory of Sichuan Province, Hospital of Chengdu University of Traditional Chinese Medicine, Chengdu, China; 2 School of Clinical Medicine, Chengdu University of Traditional Chinese Medicine, Chengdu, China; 3 Xingwen County Hospital of Traditional Chinese Medicine, Yibin, China; 4 School of Basic Medicine, Chengdu University of Traditional Chinese Medicine, Chengdu, China; 5 School of Pharmacy, Chengdu University of Traditional Chinese Medicine, Chengdu, China

**Keywords:** autophagy, gastric, cancer, phytochemicals, signal transduction, stomach neoplasms

## Abstract

Gastric cancer (GC) development follows a sequential and complex pathophysiological process known as Correa’s cascade, progressing from chronic gastritis (active or inactive) to precancerous lesions (intestinal metaplasia (IM) and dysplasia) and ultimately to adenocarcinoma. Current GC therapies have limited efficacy and high recurrence rates. Autophagy, a cellular self-degradation mechanism that removes damaged or harmful components, plays a critical role in maintaining cellular homeostasis. Phytochemicals, owing to their low toxicity and multi-target pharmacological properties, exhibit anti-inflammatory, antimicrobial, antioxidant, and anticancer activities, and certain phytochemicals have shown potential in suppressing GC progression. In the inflammatory stage, metabolites such as berberine (an alkaloid) and chlorogenic acid (a phenolic acid) modulate autophagy by regulating the phosphoinositide 3-kinase (PI3K)/AKT/mTOR pathway: upstream activation of PI3K leads to AKT phosphorylation, which subsequently inhibits the downstream autophagy-related genes Unc-51-like kinase (*ULK1*) and Beclin-1, while these phytochemicals can reverse this inhibition to reduce inflammatory cytokine secretion and promote ulcer healing. In the precancerous stage, agents such as ginsenoside Rg3 and astragaloside IV (terpenoids) regulate autophagy through the PI3K/AKT axis and by influencing p62/sequestosome 1 (SQSTM1), an autophagy adaptor that links upstream microtubule-associated protein 1 light chain 3 (LC3)-interacting partners to downstream ubiquitinated substrates—thereby restoring autophagic flux and preventing malignant transformation. In the cancer stage, phytochemicals from various subclasses (phenolics, alkaloids, and terpenoids) target the core autophagy machinery: they suppress upstream PI3K/AKT/mTOR signaling (with mTOR directly inhibiting the initiation complex formed by *ULK1*, *ATG13*, and FIP200), induce the expression of Beclin-1 and *ATG* family proteins, promote the conversion of LC3-I to LC3-II, and enhance autophagosome formation. Consequently, these metabolites inhibit GC cell proliferation, migration, and angiogenesis while inducing apoptosis and increasing chemosensitivity. From a clinical translation perspective, the consistent observation that pharmacological inhibition of cytoprotective autophagy enhances phytochemical-induced apoptosis suggests a rational combination strategy: pairing a phytochemical that induces cytoprotective autophagy with an approved autophagy inhibitor such as hydroxychloroquine. This approach has been tested *in vitro* for curcumin, quercetin, and berberine but not yet in animal models or clinical trials. Given the established safety profiles of these agents individually, such combinations could be rapidly tested in preclinical gastric cancer models and, if successful, progressed to early-phase clinical trials as adjuncts to standard chemotherapy. Moreover, ongoing clinical trials of berberine for gastrointestinal metaplasia and of resveratrol for chemotherapy toxicity reduction provide existing clinical frameworks onto which autophagy endpoint measurements could be incorporated. These findings highlight the potential of autophagy-targeting phytochemicals as promising strategies for GC prevention and treatment, offering valuable insights for clinical translation. Moreover, targeting autophagy with phytochemicals represents a promising avenue for future drug development, potentially enabling personalized combination therapies to overcome chemoresistance and improve long-term outcomes for patients with gastric cancer.

## 1 Introduction

GC represents a significant global health issue, ranking as the fifth most prevalent cancer and the second leading cause of cancer-related mortality on a worldwide scale. According to Global Cancer Statistics 2022, there will be 968,350 new cases of GC and 659,853 deaths from GC worldwide in 2022 (Smyth et al., 2020). China exhibits a high prevalence of GC, characterized by a gradual progression and subtle early symptoms, and most patients have already progressed to the advanced stage by the time it is detected, highlighting the limitations of current GC diagnosis and treatment. Although existing treatment options can improve the prognosis of GC patients, they are still characterized by strong adverse reactions, high recurrence rates, and a lack of effective means of prevention and inhibition (Bray et al., 2024). The development of GC is a multi-stage and multi-pathological process that includes chronic non-atrophic gastritis, atrophic gastritis, precancerous gastric lesions such as intestinal metaplasia (IM) and atypical hyperplasia, and gastric adenocarcinoma, collectively known as Correa’s cascade (Correa and Shiao, 1994). Therefore, inhibiting or reversing this process by targeting Correa’s cascade is key to preventing and treating GC.

Autophagy’s role in inflammation, epithelial–mesenchymal transition (EMT), and cancers has been extensively studied (Chung et al., 2020). Autophagy is a highly conserved cellular process characterized by self-degradation mechanisms that sequester proteins or damaged organelles and deliver them to lysosomes for breakdown, thereby promoting the renewal of cellular molecules, removing damaged organelles and other deleterious factors, and providing energy for cell viability.^7^ It has now been demonstrated that the autophagy pathway is an effective therapeutic target across phases of inflammation, precancerous disease, and cancer (Diaz et al., 2018). It has also been shown that autophagy has dual regulatory effects on cancer cells, promoting cancer cell survival while inhibiting cancer cell growth, reproduction, and migration, thereby suppressing cancer development (Levy et al., 2017). The PI3K/AKT/mTOR pathway is a central regulator of autophagy. Upstream signals such as receptor tyrosine kinases (RTKs) (epidermal growth factor receptor (EGFR) and HER2) and Ras activate PI3K, which converts phosphatidylinositol 4,5-bisphosphate (PIP2) to phosphatidylinositol-trisphosphate (PIP3) (Correa and Shiao, 1994; Chung et al., 2020; Diaz et al., 2018), leading to AKT activation. Activated AKT phosphorylates and inhibits tuberous sclerosis complex 1/2 (TSC1/2), allowing Rheb to activate mTOR complex 1 (mTORC1). mTORC1 phosphorylates ULK1 and ATG13, thereby blocking autophagy initiation. Downstream effectors include p70S6K and 4E-BP1. The mitogen-activated protein kinase (MAPK) pathway (extracellular signal-regulated kinase (ERK), c-Jun N-terminal kinase (JNK), and p38 mitogen-activated protein kinase (p38)) also modulates autophagy: ERK can phosphorylate ULK1 directly; JNK disrupts the B-cell lymphoma 2 (Bcl-2)–Beclin-1 complex to promote autophagy; p38 has context-dependent effects. Crosstalk between these pathways determines the net autophagic outcome. Most clinical treatments for GC are still carried out through surgery and chemotherapy, which may still lead to cancer recurrence, so targeted autophagy therapy has become a novel clinical treatment for the GC process.

It has been demonstrated that phytochemicals (such as resveratrol, curcumin, and berberine) are non-toxic, naturally occurring medicinal substances that can interact with apoptosis while also modulating autophagy by influencing autophagy-related proteins and signaling pathways such as Beclin-1, autophagy-related (*ATG*) proteins, adenosine 5’-monophosphate-activated protein kinase (AMPK), PI3K/AKT/mTOR, p38, protein 53 (p53), and LC3 (Lu and Zeng, 2025; Guo et al., 2026). These phytochemicals provide new approaches to GC treatment by targeting autophagy and playing different roles during the inflammatory, precancerous, and cancerous stages of Correa’s cascade (Braicu et al., 2022). In this overview, the mechanisms by which multiple phytochemicals target autophagy during the three stages of Correa’s cascade are summarized to elucidate their role in modulating the evolution of this pathological process. Furthermore, the shared molecular pathways through which phytochemicals modulate autophagy have been systematically summarized.

## 2 Methods

PubMed, Web of Science, Embase, and CNKI databases were searched for papers published between 1990 and May 2024. The search strings combined keywords for (“phytochemicals” OR “natural products” OR “plant metabolites”) AND (“autophagy”) AND (“gastric cancer” OR “gastritis” OR “gastric precancerous lesions” OR “Correa’s cascade”). Inclusion criteria were as follows: (a) original research articles reporting effects of defined phytochemicals (purified compounds or characterized extracts) on autophagy in gastric cancer, gastritis, or precancerous lesions; (b) studies published in English or Chinese with an English abstract; and (c) *in vitro*, *in vivo*, or clinical studies. Exclusion criteria were as follows: (a) studies without full text; (b) duplicates; (c) studies using uncharacterized extracts without any chemical analysis; and (d) reviews, editorials, or conference abstracts. A total of 581 records were screened after deduplication. Supplementary Figure S1 (Supplementary Material) shows the PRISMA flow diagram.

### 2.1 Evidence hierarchy and quality assessment

To interpret the findings, we stratified studies by evidence level. Among the included studies, no randomized controlled trials evaluating phytochemical-based autophagy modulation in patients with gastric cancer were identified. Three small human studies (two using berberine-containing botanical formulations and one using curcumin) provided safety data but did not measure autophagy markers in gastric tissue. Approximately 75% of the studies were *in vitro* experiments using established gastric cancer cell lines (AGS, MKN-45, SGC-7901, and MGC-803) or primary epithelial cells. Approximately 20% were animal studies, mostly acute chemical-induced models (ethanol, indomethacin, and MNNG). The remaining 5% were isolated mechanistic studies (molecular docking and enzyme assays). Therefore, the present review is predominantly based on preclinical evidence. Mechanistic conclusions drawn from *in vitro* studies should be considered hypothesis-generating rather than definitive.

## 3 Overview of autophagy mechanisms and its role in Correa’s cascade of GC

The autophagy process can be categorized into several phases: initiation, phagophore expansion, autophagosome formation, fusion, degradation, and recycling, as shown in Figure 1 (Xu X. et al., 2019). With the stimulation of a variety of cellular stress conditions such as starvation, hypoxia, infection, senescence, or oxidative stress, the expression of mTOR signaling and its phosphorylation is repressed and initiates autophagy by activating Unc-51-like kinase (ULK)1 and Unc-51-like kinase 2 (*ULK2*) (Togano et al., 2021). Subsequently, the PIK3C3 complex recruits effector proteins for phagophore cell membrane nucleation (Cao et al., 2019). The microtubule-associated protein 1 LC3 conjugation system and the *ATG12–ATG5–ATG16* mediate phagophore elongation and expansion (Klionsky and Schulman, 2014). The lysosomal membrane proteins lysosomal-associated membrane protein (LAMP)2 and GTPase Ras-related protein 7 (RAB7) are involved in the autophagosome fusing with lysosomes and the degradation of cargoes by a series of hydrolases (Zhou et al., 2013), and endogenous LC3-II is eventually broken down by lysosomal hydrolases. The materials recovered after degradation can be returned to the cytoplasm for reuse (Greenfield and Jones, 2013).

**FIGURE 1:**
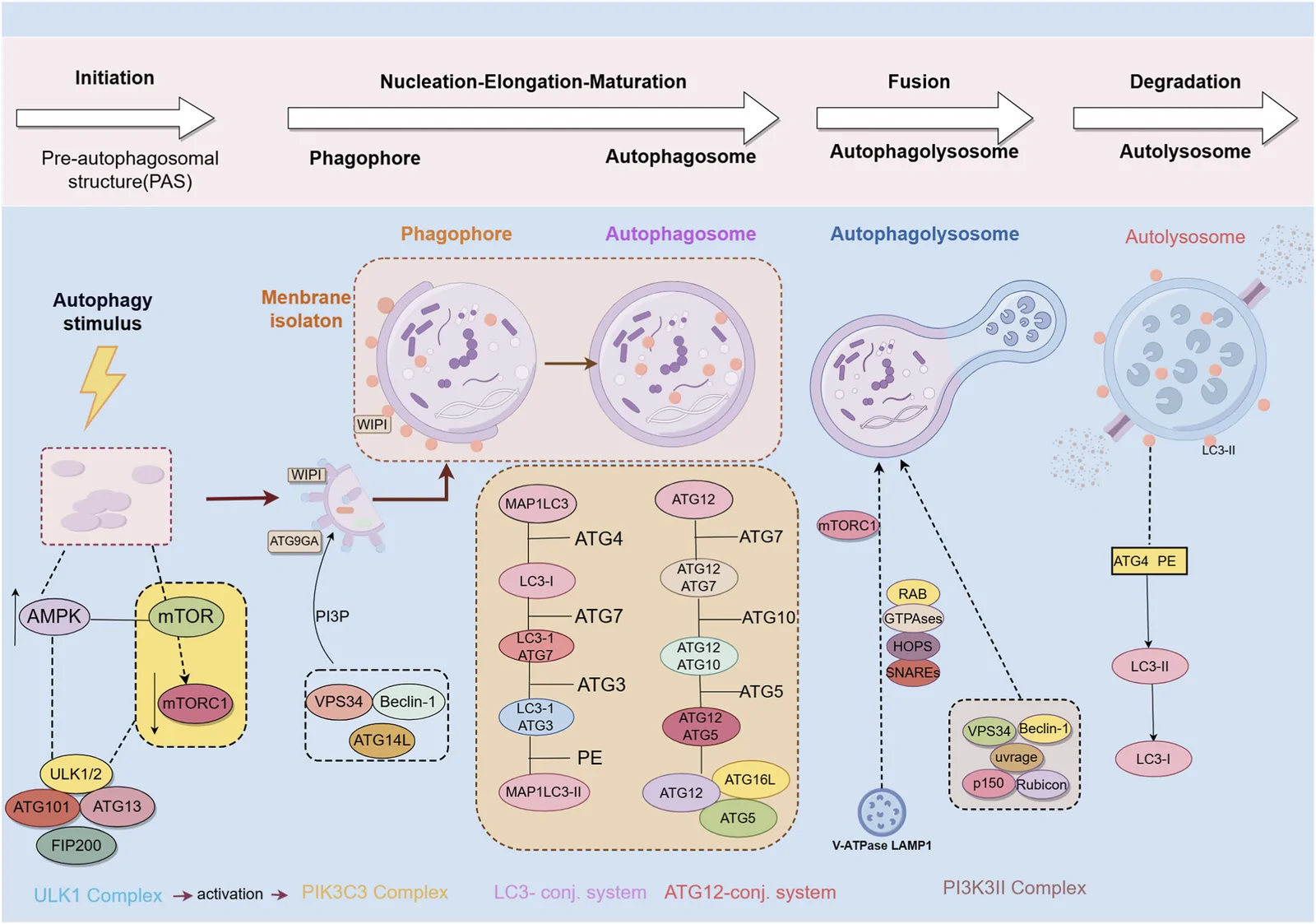
Processes and mechanisms of autophagy. Upon cellular stress (starvation, hypoxia, and ROS), mTOR activity is suppressed, leading to activation of the ULK1/2 complex. This initiates phagophore formation, followed by nucleation through the PIK3C3 complex, which produces PI3P. Elongation and closure of the autophagosome require two ubiquitin-like conjugation systems: the ATG12–ATG5–ATG16 complex and the LC3 lipidation system (involving ATG4, ATG7, and ATG3). LC3-II is incorporated into the autophagosomal membrane. Mature autophagosomes fuse with lysosomes (facilitated by LAMP2 and RAB7), where cargoes are degraded by hydrolases. Degraded products (amino acids and fatty acids) are recycled to the cytoplasm. Key regulatory nodes (mTOR, ULK1, Beclin-1, LC3, and p62) are highlighted.

### 3.1 Autophagy in the inflammatory phase

Chronic infection with *Helicobacter pylori* (*H. pylori*) is the predominant cause of atrophic gastritis. The gastric epithelium can be activated through *H. pylori* and its virulence toxins (Busada et al., 2019). Cytotoxin-associated gene A limits the autophagosome production, decreases the autophagy marker LC3-II’s expression, promotes the accumulation of SQSTM1 (Sequestosome 1), and increases the expression levels (Li N. et al., 2017). Autophagy suppresses inflammatory responses by modulating inflammatory signals, which, in turn, prevents interferon regulatory factor 3 (IRF3) activation and NOD-like receptor family pyrin domain containing 3 (NLRP3) inflammatory vesicle formation (Deretic, 2021). The autophagy factor *ATG16L1* (autophagy-related 16-like 1) increases the production of interleukin (IL)-8 in gastric epithelial cells, inhibits *H. pylori*-induced autophagy, improves the endoplasmic reticulum (ER), and lowers tumor necrosis factor alpha (TNF-α) expression levels (Mommersteeg et al., 2022). In *H. pylori-*induced inflammatory responses, reactive oxygen species (ROS) levels have been elevated. ROS can control autophagy *via* the MAPK/JNK/mTOR pathway (Bhattacharyya et al., 2014).

### 3.2 Autophagy in the precancerous phase

The development of gastric precancerous lesions (GPLs) is associated with inflammatory response, glycolysis, and apoptosis. Research has indicated a correlation between glycolysis and the development of precancerous gastric lesions, while deficiencies in autophagy have been found to promote glycolytic activity. Hypoxia-inducible factor 1 (HIF-1) activates transcripts encoding glycolytic enzymes and glucose transporters, which in turn facilitate the development of GC. HIF-1 induces lactate dehydrogenase A (LDHA) and monocarboxylate transporter 4 (MCT4) to regulate lactic acid production and promote the transformation of pre-cancerous lesions to cancers. When mTOR activates mTOR complex 1 (mTORC1) (Qin et al., 2015), the translation rate of HIF-1α mRNA increases, thereby promoting the activation of glycolytic pathways (Semenza, 2010). The above findings show that autophagy can inhibit precancerous lesions, thereby preventing the development of GC (Cai et al., 2019).

### 3.3 Autophagy in the GC phase

Autophagy plays two distinct roles in the progression of cancer. First, in advanced stages, autophagy helps the tumor survive various stresses and encourages tumor metastasis and recurrence. Cancer cells can eliminate damaged mitochondria and evade apoptosis through mitochondrial autophagy, while autophagy-associated proteins such as p62 and p53, autophagy-related (*ATG*) proteins, *BECN1* (Beclin-1), and the neighbor of BRCA1 gene 1 (NBR1) accumulate when autophagy is defective (Ishaq et al., 2020). Furthermore, autophagy suppression facilitates glycolysis and the malignant progression of GC and enhances EMT and metastasis in GC (Pang et al., 2021). Tumor angiogenesis is promoted when Beclin-1 expression is inhibited in SGC-7901 cells (Ding et al., 2014).

Second, autophagy suppresses tumor activity by preventing DNA damage, ROS, tissue damage, inflammation, and other factors that induce tumor growth. Several genes linked to autophagy, including *ATG* proteins, tuberous sclerosis complex subunits 1 and 2 (*TSC1–TSC2*), and *TP53/p53*, have been identified in gastric cancer cells and shown to induce autophagy and suppress GC progression (Yang and Klionsky, 2020). Autophagy can also prevent EMT by targeting signaling pathways such as PI3K/AKT/mTOR and Janus kinase signal transducer and activator of transcription (JAK/STAT), along with EMT-related transcription factors (Chen et al., 2019). In summary, autophagy is a complex process in GC that can promote cancer cell survival and proliferation while inhibiting their ability to invade and metastasize. This information can be used to develop novel therapeutic approaches against GC (Camuzard et al., 2020). The structures of phytochemicals are shown in Figure 2.

**FIGURE 2:**
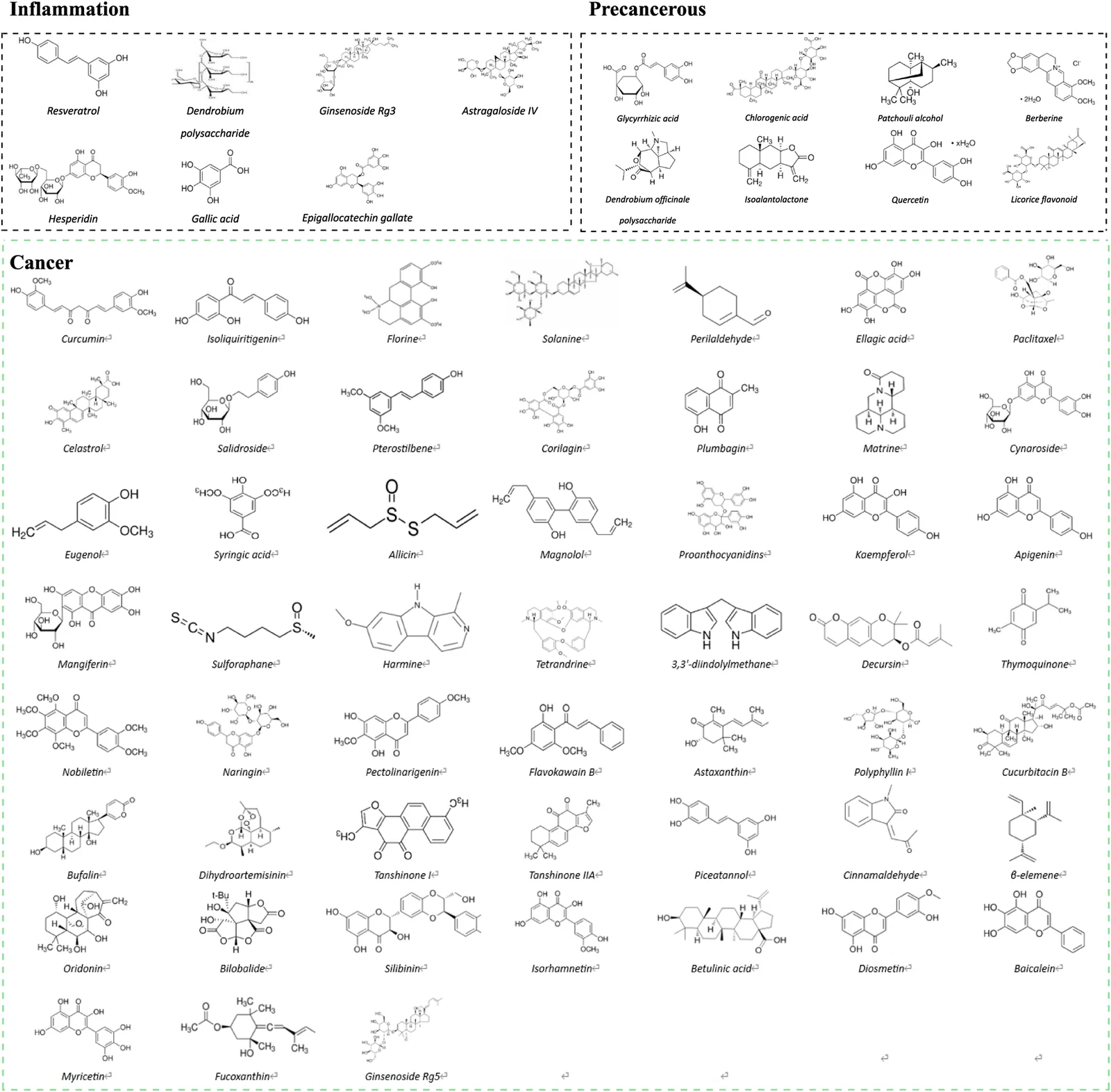
Structures of phytochemicals in Correa’s cascade.

## 4 Autophagy modulation by phytochemicals via key signaling pathways

### 4.1 Gastritis phase

During the gastritis phase, phytochemicals primarily exert gastroprotective effects by suppressing inflammatory responses, mitigating oxidative stress, and preserving gastric mucosal integrity. Mechanistically, these metabolites modulate key autophagy-related signaling cascades, including the MAPK and PI3K/AKT/mTOR pathways, to limit excessive inflammation and promote mucosal healing. The following sections categorize these phytochemicals based on their primary molecular targets, as summarized in Table 1.

**TABLE 1:** Mechanisms of phytochemical-mediated autophagy targeting in Correa’s cascade.

Phytochemical	Origin	Experiment model	Mechanism of action
Glycyrrhizin	*Glycyrrhiza uralensis* Fisch’s root	AGS	Increases the expression of LC3-II and LAMP1
Chlorogenic acid	Green coffee extracts and tea	Gastric mucosa	Activates Nrf-2 to improve the expression of Beclin-1 and *Atg5*/7/LC3-II
		Indomethacin-induced gastric ulcer rat model	Activation of AKT/mTOR inhibits autophagy overexpression
		ST-infected mouse model	Activates lncRNA and GAS5/mir-23a/PENT and p38 MAPK
Dendrobium kimura polysaccharide	*Dendrobium officinale* Kimura & Migo	Gastric mucosal injury rat model	Activates AMPK and inhibits p62 and mTOR
Patchouli alcohol	*Pogostemon cablin* (Blanco) Benth.	Ethanol-induced rat mod	Inhibits the phosphorylation of p38, JNK, and ERK
Incarvillea compacta Maxim	Incarvillea and Bignoniaceae	*In vivo* mouse acute gastritis model	Inhibits the PI3K/AKT pathway
Berberine	*Coptis chinensis Franch.*	MGC-803	Inhibits the phosphorylation of p38, MAPK, ERK1/2, and JNK
Isoalantolactone	*Inula helenium* Hook.f.	Human gastric epithelial cells	Inhibits MAPK/PI3K/AKT
Licorice flavonoid	*Glycyrrhiza uralensis* Fisch. ex DC.	GES-1	Activates the *EGFR*/ERK pathway
Quercetin	*Allium cepa* L.; *Asparagus officinalis* L.; *Lactuca sativa* L.	H_2_O_2_-induced GES-1 cells	Activates the PI3K/AKT pathway
Resveratrol	Grape wine	GES-1, AGS, BGC-823, SGC-7901	Inhibits the PI3K/AKT pathway to activate *FoxO4*
Celastrus orbiculatus	Stem of the Chinese herbal *C. orbiculatus*	MNNG-induced PLGC rat model	Upregulated PDCD4 to inhibit *ATG5* expression
		MGC-803	Inhibits Lgr5 expression to reverse EMT
Hesperidin	*Citrus reticulata* Blanco fruits	Rat model of long-term MNNG exposure	Reverses the MNNG-induced decrease in PI3K/AKT, Beclin-1, LC3-II, and *ATG5*
Epigallocatechin gallate	*Camellia sinensis* L	Rat gastric mucosa	Inhibits PI3K/AKT/mTOR
Ginsenoside Rg3	*Panax ginseng*	ATP4a−/− mouse model	Inhibits PI3K/AKT/mTOR to inhibit glycolysis
Astragaloside IV	Astragali Radix’s roots	MNNG-induced rat GPL model	Upregulated *p53* expression to activate miRNA-34a and inhibit LDHA expression
		Gastric mucosa of Mon-induced GPL rats	Downregulation of *p53* expression inhibits the overexpression of Ambra1 and Beclin-1 mRNA
*D. officinale* polysaccharides	*Dendrobium officinale* Kimura & Migo	MNNG-induced GPL rat model	Inhibits the Wnt/β-catenin pathway
*Panax notoginseng saponins*	*Panax notoginseng* (Burkill) F. H. Chen ex C. H. Chow.	N-Nitroso-N-methylurea-induced GPL rat model	Inhibits the PI3K/AKT/mTOR pathway
Resveratrol	Grapes, wine	SGC-7901	Inhibits the AKT/mTOR pathway and activates the p38 MAPK/ERK pathway
		HGC-27	Accumulates LC3-II through the inhibition of dihydroceramide desaturase
		SGC-7901/DOX	Inhibits the PI3K/AKT/p70S6K pathway
Curcumin	*Curcuma longa Linn.*	AGS	Inhibits PI3K/AKT/mTOR
		BGC-823, SGC-7901, MKN-28	Increased expression of AVOs, LC3-II, p53, and *ATG* proteins
Isoliquiritigenin	*Glycyrrhiza uralensis* Fisch’s root	MKN-28	Increases the expression of LC3-II and Beclin-1 and inhibits p62
Isoliquiritigenin-17	*Glycyrrhiza uralensis* Fisch’s root	SGC-7901	Inhibits AKT/mTOR, increases the expression of LC3-II and Beclin-1, and inhibits p62
Magnoflorine	*Coptis chinensis* Franch.	SGC-7901, BGC-823, MGC-803	Inhibits AKT/mTOR and upregulates the expression of LC3-II, p62, LAMP1, and JNK
Solanine	*Solanum genus*	Human gastric epithelial cell lines GES-1, SGC-7901, and MGC-803	Inhibits the overexpression of AAMDC/AFT4/MYC/Sesn2 to improve LC3-II/LC3-I
Celastrol	*Tripterygium wilfordii* Hook. f.	AGS, YCC	Activates AMPK and inhibits the AKT/mTOR pathway
Salidroside	*Rhodiola rosea* Linn.	AGS	Inhibit the PI3K/AKT/mTOR pathway
Pterostilbene	Blueberries	N87, MKN-45	Accumulates AVOs, improves LC3-II, and inhibits p62
Celastrus orbiculatus	Stem of the *Celastrus orbiculatus* Thunb.	MGC-803/mTOR cells	Inhibits AKT/mTOR, accumulates AVOs, and increases the expression of LC3-II and Beclin-1
Corilagin	*Caesalpinia coriaria*	SGC-7901, BGC-823	Accumulates AVOs and improves the expression of LC3-II
		N-methyl-N′-nitro-N-nitrosoguanidine stimulated gastric cancer rats	Inhibits the PI3K/AKT pathway
Perillaldehyde	*Perilla frutescens*	MFC, GC9811-P	Improves the expression of Beclin-1, LC3-II, and AMPK and accumulates AVOs
Alpha, 2′-dihydroxy-4,4′-dimethoxydihydrochalcone	Meliaceae Juss.	MKN-45	Increases the expression of LC3-II, *ATG5*, *ATG7*, and Beclin-1
Plumbagin	Plumbaginaceae, Droseraceae, and Ebenaceae	MKN-28	Increases the expression of LC3-II and Beclin-1
Syringic acid	*Alpinia calcarata* Roscoe	AGS	Inhibits the AKT/mTOR pathway
Eugenol	*Syringa oblata* Lindl.	Rat gastric cancer model	Inhibits NF-kB and increases the expression of p21 and p53
Allicin	*Allium sativum* L.	MGC-803, BGC-823, SGC-7901	Inhibits the PI3K/AKT pathway and p38
Magnolol	*Houpoea officinalis*	SGC-7901	Inhibits the PI3K/AKT pathway
Urolithin A	Ellagitannins and ellagic acid	HGC-27, MKN-45	Inhibits the PI3K/AKT/mTOR pathway
Mangiferin	Mango fruits and leaves	SGC-7901	Inhibit the PI3K/AKT/mTOR pathway
Sulforaphane	Cruciferous vegetables	SGC-7901	Activates miR-4521/PIK3R3 to inhibit the expression of LC3-II and Beclin-1
Harmine	*Peganum harmala* L.	MGC-803, SGC-7901	Activates AMPK and improves *ULK1*
Tetrandrine	*Stephania tetrandra*	HGC-27	Inhibits the AKT/mTOR/PS6K/P-4EBP1 pathway
Matrine	*Sophora flavescens* Ait’s root and stem	SGC-7901	Inhibits the AKT/mTOR pathway and increases the expression of LC3-II and Beclin-1
		BGC-823, AGS, SGC-7901	Impairing the activities of lysosomal proteases
Chinese dragon’s blood ethyl acetate extract	The red resin of Dracaena cochinchinensis (Lour.) S. C. Chen	MGC-803, HGC-27	Inhibits mTOR and increases the expression of Beclin-1, *ATG5*, *ATG3*, and LC3-II
Nobiletin	*Citrus reticulata* Blanco fruits	SNU-16	Inhibits the AKT/mTOR pathway and p62
Naringin	*Citrus reticulata* Blanco fruits	AGS	Activates AMPK and inhibits the PI3K/AKT/mTOR pathway
		SUN-1	Inhibition of PI3K/AKT-induced autophagy to promote apoptosis
Pectolinarigenin	*Cirsium* *chinense* and citrus fruits	AGS, MKN-28	Inhibits the PI3K/AKT/mTOR pathway
		AGS-xenograft BALB/c nude mice	Inhibits PI3K/AKT/mTOR
Flavokawain B	Roots of *Piper methysticum* G. Forst.	AGS-transplanted nude mouse model	Inhibit PI3K/AKT/mTOR and reduce the expression of Bcl-2 to inhibit its inhibition of Beclin-1
Kaempferol	Roots of *Kaempferia galanga* Linn.	AGS SNU-216, NCI-N87, SNU-638, MKN-74	Activates AMPK/*ULK1* and IRE1–JNK–CHOP and inhibits G9a in gastric cancer cells
Bufalin	Chansu	MGC-803	Inhibits PI3K/AKT/p70S6K and activates the ER signal
		SGC-7901, BGC-823	Activates the IRE1–JNK pathway
Eriodictyol	*Reynoutria japonica* Houtt.	AGS, HGC-27, MKN-45, GES-1	Inhibits PI3K/AKT
Black ginger-selenium nanoparticle	Kaempferia parviflora	AGS	Inhibits PI3K/AKT/mTOR
Dihydroartemisinin	*Artemisia capillaris* Buch. -Ham. ex Roxb.	SGC-7901	Inhibits PI3K/AKT
		BGC-823	Activates ERK1/2, ′JNK1/2, and p38 MAPK
Tanshinone I	*Salvia miltiorrhiza* Bunge	BGC-823, SGC-7901	Inhibits the negative regulation of Beclin-1 by Bcl-2 and increases LC3-II/LC3-I
Tanshinone IIA	*Salvia miltiorrhiza* Bunge	SNU-719, SUN-719R, SUN-620	Increases the expression of LC3B-II and inhibits p62
Paclitaxel	*Taxus wallichiana* var. *chinensis (Pilg.)Florin*	AGS, BGC-823	Increases the expression of LC3B-II, SQSTM1, PI3KII, *ATG* proteins, and Beclin-1 and inhibits p62 and mTOR
Oridonin	*Rabdosia* (Blume) Hassk.	HGC-27, MGC-803, AGS	Increases the expression of LC3B
Jaridon 6	*Rabdosia* (Blume) Hassk.	MGC-803	Inhibits the STRT1 enzyme by inhibiting PI3K/AKT
Bilobalide	*Ginkgo biloba*	AGS, N-Nitroso-N-methylurea-induced rat gastric cancer model	Decreases the expression of ERK 1/2, p38, and JNK1/2, and inhibits the phosphorylation of PI3K1/2 and AKT proteins
Silibinin	S*ilybum marianum.*	SGC-7901	Inhibits the expression of p-p38 MAPK
		SNU226, SNU668	Inactivation of the MEK/ERK pathway inhibits TNF-α-induced MMP-9 expression
Isorhamnetin	*Hippophae rhamnoides* Linn., *Ginkgo biloba* L., *Tamarix ramosissima* Ledeb.	HGC-27	Inhibits MAPK/mTOR
		MKN-45	Inhibits the phosphorylation of PI3K/AKT/mTOR and the expression of Beclin-1 and LC3B
Cynaroside	*Cynara scolymus*	HGC-27, MKN-45, SGC-7901	Inhibits MET levels to reduce the phosphorylation of PI3K/AKT/p70S6K
Myricetin	*Morella rubra* Lour.	AGS	Inhibits the phosphorylation of PI3K/AKT/mTOR
Fucoxanthin	Brown seaweeds	SGC-7901	Accumulates AVOs and increases the expression of Beclin-1 and LC3-II
Berberine	*Coptis chinensis Franch.*	BGC-823	Inhibits the expression of MAPK/mTOR/p70S6K/AKT
		SGC-7901/DDP BGC-823/DDP	Inhibits the PI3K/AKT/mTOR pathway
Astaxanthin	Lobsters	AGS	Activates AMPK and inhibits AKT/mTOR
Polyphyllin I	*Paris polyphylla* Smith.	HGC-27	Inhibits the phosphorylation of PI3K/AKT/mTOR
Apigenin	Celery, parsley, thyme, chamomile, onion	AGS	Inhibits PI3K/AKT/mTOR
		SUN-638	Activates AMPK/*ULK1*, inhibits mTOR, and activates ER stress
Ginsenoside Rg5	*Panax ginseng* C. A. Mey.	SGC-7901, BGC-823	Increases the generation of ROS and activates MAPK
Quercetin	*Allium cepa* L.; *Asparagus officinalis* L.; *Lactuca sativa* L.	AGS, MKN-28	Inhibits the AKT/mTOR/HIF-1α pathway
Astragaloside IV	Roots of *Astragalus propinquus* Schischk.	BGC-82, MKN-74	Inhibits the activation of the PI3K/AKT/NF-kB pathway to inhibit EMT
Trichosanthin	Root tuber of *Trichosanthes* L.	MKN-45	Increases the production of ROS and promotes the expression of p-NF-kB and p-p53
Betulinic acid	*Betula*, *Platanus* L., and *Eucalyptus* L'Hér.	SGC-7901	Inhibits ERK/EMK/p62 and accumulates AVOs
Thymoquinone	*Nigella sativa* L.	MGC-803, SGC-7901	Inhibition of the PI3K/AKT/mTOR pathway and the EMT process
Diosmetin	*Citrus reticulata* Blanco fruits	HGC-27	Activates the AMPK/JNK pathway and increases the phosphorylation of ERK1/2, p28, and JNK
A-24	*Allium* L. species.	AGS, SGC-7901	Inhibits PI3K/AKT/mTOR
		*p53* wild-type or p53-deletion AGS cells	Promotes the production of ROS and inhibits PI3K/AKT/mTOR
Baicalein	Roots of *Scutellaria baicalensis*	SGC-7901/DDP	Inhibits the phosphorylation of AKT/mTOR/p62
		MGC-803	Inhibits the phosphorylation of PI3K/AKT
Proanthocyanidins	Grape seeds	MGC-803, SGC-7901	Inhibits the phosphorylation of PI3K/AKT/mTOR
Piceatannol	Red wine, grapes	SGC-7901, BGC-823, MKN-28, MGC-803, HGC-27, AGS	Enhances the effect of Beclin-1
Cinnamaldehyde	*Cinnamomum cassia*	SNU-638, SNU-216, AGS, NCI-N87, MKN-45, MKN-74	Increases the phosphorylation of AMPK/*ULK1* and inhibits the phosphorylation of mTOR
Decursin	*Angelica sinensis*.	NU-216, NCI-N87	Inhibition of cathepsin C (CTSC) expression induces autophagy dysregulation
Cucurbitacin B	Cucurbitaceae Juss. plants.	SGC-7901/DDP	Inhibits the CIP2A/PP2A/mTORC1 signaling pathway
β-elemene	Rhizoma zedoariae	MGC-803, SGC-7901	Inhibits PI3K/AKT/mTOR/p70S6K1 phosphorylation
3,3′-diindolylmethane	Cruciferous vegetables	BGC-823, SGC-7901	Inhibits the expression of miR-30e-*ATG5*
​	​	BGC-823, SGC-7901	Upregulation of AMPK/STIM1/ATF6 expression induces ER stress

Abbreviations: AMPK, adenosine 5′-monophosphate-activated protein kinase; ATG, autophagy-related protein; AVO, acidic vesicular organelle; CQ, chloroquine; EMT, epithelial–mesenchymal transition; ERK, extracellular signal-regulated kinase; GPL, gastric precancerous lesion; HIF-1α, hypoxia-inducible factor 1α; JNK, c-Jun N-terminal kinase; LC3, microtubule-associated protein 1 light chain 3; MAPK, mitogen-activated protein kinase; mTOR, mammalian target of rapamycin; NF-κB, nuclear factor kappa B; Nrf2, nuclear factor erythroid 2-related factor 2; p53, tumor protein p53; p62/SQSTM1, sequestosome 1; PI3K, phosphoinositide 3-kinase; PLGC, precancerous lesions of gastric cancer; ROS, reactive oxygen species; ULK1, Unc-51-like kinase 1.

#### 4.1.1 MAPK signaling pathway (p38, ERK, and JNK)

##### 4.1.1.1 Sesquiterpenoids

Patchouli alcohol is the key constituent of the traditional Chinese medicine *Pogostemon cablin* (Blanco)Benth., a natural tricyclic sesquiterpenoid. The study showed that patchouli alcohol increased gastric prostaglandin E2 and non-protein sulfhydryl group levels, enhanced the mRNA expression of cyclooxygenase 1/2 (COX-1 and COX-2), regulated the levels of IL-6, IL-10, and TNF-α, restored the levels of glutathione (GSH), catalase (CAT), and malondialdehyde (MDA), and ameliorated gastric ulcers caused by ethanol and indomethacin (Zheng et al., 2014).

Isoalantolactone is a sesquiterpene-lactone metabolite. It is extracted from the root of *Inula helenium* Hook. f. & Thomson. Studies have shown that in zebrafish models of inflammation, isoalantolactone reduces ROS accumulation, blocks the MAPK and PI3K/AKT signaling pathways and their related factors, inhibits inflammation, protects the gastric mucosa, and reduces ethanol-induced gastric ulcer (Zhou et al., 2023).

##### 4.1.1.2 Flavonoids

Licorice flavonoid is a flavonoid compound extracted from the conventional plant *Glycyrrhiza uralensis* Fisch. ex DC. Studies have shown that, in ethanol-treated GES-1 cells, licorice flavonoids increase cell viability at concentrations ranging from 2.5 to 5 μg/mL. An animal experiment demonstrated that *EGFR*, p-c-Raf, c-Raf, and *Hras* proteins, along with p-ERK/ERK ratios, were elevated in licorice flavonoid-treated GES-1 cells, ultimately leading to gastric mucus cell regeneration. These results indicate that licorice flavonoids can activate the *EGFR*/ERK pathway and protect the gastric mucosa (Wu et al., 2023).

##### 4.1.1.3 Alkaloids

Berberine (BER) is one of the isoquinoline quaternary alkaloids extracted from *Coptis chinensis Franch.*, which has anti-inflammatory, antibacterial, anti-cancer, and other biological activities. It has been shown that BER mediates the inflammatory response through autophagy (Samadi et al., 2020; Xiong et al., 2022). Treatment of MGC-803 cells with different concentrations of BER suppressed GC cell proliferation, while IL-8 production and the phosphorylation of p38, MAPK, ERK1/2, and JNK were downregulated. These findings suggest that BER can inhibit GC progression by reducing IL-8 production and MAPK pathway activation (Li et al., 2016).

#### 4.1.2 PI3K/AKT/mTOR signaling pathway

##### 4.1.2.1 Polyphenols

Chlorogenic acid (CGA) is one of the most common polyphenolic chemicals found in the human diet. The role of CGA-mediated autophagy is twofold: first, it can promote the initiation and progression of autophagy by activating the nuclear factor erythroid 2-related factor 2 (Nrf2) signaling pathway. Second, it can protect the gastric epithelium, enhance cellular autophagy, and scavenge ROS and other damaging factors by upregulating autophagy-related proteins, such as Beclin-1, LC3-II, and *ATG5*. In addition to the above findings, in an indomethacin-induced gastric ulcer model, CGA treatment ameliorated indomethacin-induced gastric tissue damage in rats and reduced the elevated ulcer index and expression levels of LC3-II/LC3-I, Beclin-1, and p62. Further research showed that CGA restored the reduced AKT/mTOR signaling induced by indomethacin and increased the Bcl-2/Bax (Bcl-2-associated x protein) ratio (Liu et al., 2022). The above results indicated that in the indomethacin-induced mouse model, CGA inhibited excessive autophagy and restored the autophagy pathway to normal levels, thereby helping reduce damage to gastric epithelial cells (Ahmed et al., 2021).

##### 4.1.2.2 Polysaccharides

Dendrobium officinale polysaccharide is the major active ingredient extracted from the Chinese medicinal plant *Dendrobium officinale* Kimura & Migo, whereas Dendrobium kimura polysaccharide is a polysaccharide isolated from Dendrobium kimura (Table 1). Studies showed that based on Western blot and immunohistochemistry analyses, dendrobium kimura polysaccharide could activate the AKT/mTOR signaling pathway to increase the expression levels of AMPK, heme oxygenase-1 (HO-1), Beclin-1, LC3B, and suppress p62 and mTOR expression within gastric cells. The *in vitro* experiments indicated that in ethanol-induced GES-1 cells, dendrobium kimura polysaccharide activated the mTOR/AMPK signaling pathway, inhibited the expression of Bax and caspase-3, and increased the expression of Bcl-2, indicating that dendrobium kimura polysaccharide could inhibit the apoptosis induced by ethanol (Ke et al., 2020).

##### 4.1.2.3 Flavonoids

Quercetin is a polyphenolic flavonoid molecule metabolite. In an H_2_O_2_-induced cell model, quercetin can reduce ROS accumulation and matrix metalloproteinase (MMP) expression at 25 μM–50 μM concentrations. The research also found that it enhanced the AKT and PI3K levels. The results above imply that quercetin can activate the PI3K/AKT pathway and reduce oxidative stress-induced damage and inflammatory responses in the GES-1 cells (Yao et al., 2021).

*Incarvillea compacta Maxim* extract (ICE) is a traditional Tibetan medicinal preparation that contains flavonoids, triterpenoids, and steroids. Research has shown that, in RAW246.7 cells, ICE downregulates the expression and phosphorylation levels of phosphoinositide-dependent kinase 1 (PDK1), AKT, GSK3β, and the inflammatory factor NLRP3. In addition to that, in the HCl/EtOH-induced acute gastritis mouse model, ICE was found to improve inflammatory lesions in gastric tissue (Zhang J. et al., 2022).

Baicalein is a natural metabolite isolated from *Scutellaria baicalensis*. It has been confirmed that baicalein treatment inhibits the phosphorylation levels of p62, AKT, mTOR while upregulating the expression of LC3 and Beclin-1 in SGC-7901/DDP cells, thereby inducing cellular autophagy. It was also found that baicalein enhances the sensitivity of SGC-7901/DDP cells to DDP, demonstrating its efficacy in cancer treatment (Li et al., 2020). Additionally, baicalein induces autophagy in MGC-803 cells by reducing PI3K/AKT phosphorylation and increasing the expression of LC3-II and p62 proteins (Qiao et al., 2019).

##### 4.1.2.4 Stilbenoids

Resveratrol is a naturally occurring polyphenolic metabolite with chemopreventive effects that operates through various mechanisms. Some studies have shown that forkhead box O (FOXO) proteins regulate the proliferation of GC cells and influence cell cycle arrest through the autophagy pathway (Chen et al., 2021; Wang GJ. et al., 2015). In AGS, MKN-35, and chenodeoxycholic acid-induced GES-1 cells, resveratrol reduced *CDX2* (caudal-type homeobox transcription factor 2) expression and increased *FoxO4* Ser197 phosphorylation. Furthermore, after treatment with the PI3K/AKT pathway inhibitor LY294004, si*FoxO4*, and Lv‐*FoxO4*, *FoxO4* phosphorylation was reduced, reversing the resveratrol-induced reduction in *CDX2* expression. The study also found that the PI3K/AKT/p‐*FoxO4*/*CDX2* pathway is suppressed in IM tissues. These results indicate that resveratrol reduces the risk of bile acid-induced gastric intestinal metaplasia (GIM) by inhibiting PI3K/AKT pathway phosphorylation, which thereby activating *FoxO4* by reducing *CDX2* expression in GES-1 cells (Lu et al., 2021).

##### 4.1.2.5 Triterpene saponins

Astragaloside IV (AS-IV) is a triterpene glycoside isolated from the roots of *Astragalus propinquus* Schischk. It was found that AS-IV could improve gastric mucosal dysplasia in the 1-methyl-2-nitro-1-nitrosoguanidine (MNNG)-induced rat model and that microRNA (miRNA)-34a plays a role in the autophagy process (Sharma et al., 2020; Ilisso et al., 2018). AS-IV upregulates the expression of *p53* protein, which activates miRNA-34a activity, regulates the expression of *LDHA* (glycolysis rate-limiting enzyme), and inhibits the glycolytic pathway to suppress MNNG-induced precancerous lesions of gastric cancer (PLGC) (Zhang C. et al., 2018). Although this study has not yet assessed the changes in the expression levels of autophagy-related factors in the rat model following the activation of miRNA-34a by AS-IV, it still provides a new target for AS-IV to inhibit PLGC by targeting autophagy. The *p53* protein can inhibit the expression of anti-apoptotic genes while simultaneously promoting the expression of the pro-apoptotic proteins Bax and Beclin-1 (Mrakovcic and Frohlich, 2018). Following cellular stimulation both *in vivo* and *in vitro*, *p53* activates AMPK, thereby inhibiting mTOR activity. *p53* can also inhibit Beclin-1 degradation (Rah et al., 2021), and its upregulation leads to the activation of ULK1 and ULK2, which promote autophagy. AS-IV can also inhibit the overexpression of Ambra1 and Beclin-1 mRNA by downregulating the aberrant expression of *p53*, thereby reducing the levels of Beclin-1, p62, *ATG5–ATG12*, and LC3, decreasing autophagy activity, alleviating gastric epithelial damage, and reversing MNNG-induced rat GPL (Cai et al., 2018). AS-IV reversed the epithelial–mesenchymal transition (EMT) process in BGC-823 and MKN-74 cells by inhibiting the activity of the PI3K/AKT/NF-kB pathway and reducing the expression levels of N-cadherin and Vimentin. It also restored the transforming growth factor β1 (TGF-β1)-induced reduction in E-cadherin expression in BGC-82 and MKN-74 cell lines (Zhu and Wen, 2018).

#### 4.1.3 Other signaling pathways

##### 4.1.3.1 Triterpene saponins

Glycyrrhizic acid is a major bioactive metabolite isolated from the *Glycyrrhiza uralensis* Fisch. In glycyrrhizic acid-treated AGS and *H. pylori*-infected AGS cells, glycyrrhizic acid dose-dependently boosted the level of autophagy proteins LC3B-II and LAMP1, promoting lysosome formation and suppressing *H. pylori*-mediated AGS cell growth. Further research has shown that silencing of high-mobility group box 1 (HMGB1) increases LC3B-II expression and reduces *H. pylori* levels. This suggests that autophagic degradation may be associated with HMGB1 inhibition. The *in vitro* study also verifies that glycyrrhizic acid enhanced lysosomal degradation. Animal experiments showed that glycyrrhizic acid suppressed HMGB1 levels and regenerated *H. pylori*-infected rats’ gastric mucosal injury. So, glycyrrhizic acid, by inducing autophagic lysosome formation in infected cells, inhibited the inflammatory response and ameliorated gastric tissue damage (Khan et al., 2023).

#### 4.1.4 Summary and perspective on phytochemicals in gastritis

Collectively, the reviewed studies underscore the potential of phytochemicals to ameliorate gastritis by targeting autophagy-related pathways. A common mechanism involves the inhibition of the MAPK and PI3K/AKT signaling cascades, leading to reduced secretion of pro-inflammatory cytokines (IL-8 and TNF-α) and attenuation of oxidative stress. Metabolites such as patchouli alcohol, licorice flavonoid, and berberine demonstrate efficacy in promoting gastric ulcer healing and restoring mucosal barrier function.

However, several critical gaps remain. First, the majority of studies focus on acute gastric injury models (ethanol- or indomethacin-induced), with limited investigation of chronic *H. pylori* infection models that more accurately reflect human gastritis. Second, while the involvement of autophagy is often inferred from changes in LC3-II and Beclin-1 expression, direct evidence of autophagic flux and its causal role in the protective effects is frequently lacking. Flavonoids (licorice flavonoid, quercetin) tend to activate EGFR/ERK or PI3K/AKT pathways, promoting mucosal repair, whereas alkaloids (berberine) and sesquiterpenoids (patchouli alcohol, isoalantolactone) predominantly inhibit MAPK signaling to reduce inflammation. Polysaccharides (dendrobium) activate AMPK/mTOR. Despite these differences, all classes converge on reducing oxidative stress and cytokine release. Notably, no study has directly compared two phytochemicals from different classes under identical conditions, so relative potency cannot be inferred. Furthermore, most effective concentrations *in vitro* (quercetin 25–50 µM) far exceed reported plasma levels in humans, raising doubts about clinical relevance. Future gastritis research should prioritize chronic *H. pylori* models, autophagic flux assays, and dose selection guided by pharmacokinetic data.

### 4.2 GPL phase

In the precancerous phase, the reversal or inhibition of intestinal metaplasia and dysplasia is critical. Phytochemicals intervene at this stage by reactivating tumor-suppressive autophagy, primarily by inhibiting the PI3K/AKT/mTOR pathway and regulating glycolysis and EMT. The following section analyzes these metabolites based on their impact on pivotal pathways.

#### 4.2.1 PI3K/AKT/mTOR signaling pathway

##### 4.2.1.1 Flavonoids

Hesperidin, one of the major constituents of citrus fruits, is a naturally occurring polymethoxy flavonoid. According to some research, in the MNNG-induced rat model, hesperadin reversed the inactivation of the PI3K/AKT signaling pathway induced by MNNG, slowing down the EMT process, reversing the decreasing levels of Beclin-1, LC3-II, and *ATG5*, and activating the gastric injury rat model’s autophagy to prevent the growth of GC (Liang et al., 2022; Lu L. et al., 2023).

##### 4.2.1.2 Polyphenols

Epigallocatechin is a class of natural polyphenolic metabolites extracted from the tea tree. Research has shown that in the MNNG-induced PLGC rat model, epigallocatechin treatment could restore gastric mucosal injury and promote growth in PLGC rats, and epigallocatechin also blocked the PI3K/AKT/mTOR signaling pathway and upregulated the expression of cleaved caspase-3 and phosphatase and tensin homolog (PTEN), hindering the progression of PLGC to inhibit the development of GC (Zhu et al., 2021).

In addition to protecting the gastric mucosa, inhibiting precancerous gastric lesions is another important therapeutic application of Dendrobium polysaccharide. Research has demonstrated that with the suppression of the wingless-related integration site (Wnt)/β-catenin signaling pathway, the expression of p62 and the accumulation of transcription elongation factor b (TEFB) have been increased, indicating that the β-catenin signaling pathway can control autophagy by altering the expression of p62 and LC3 proteins (Lorzadeh et al., 2021; Ma et al., 2023). Western blot analysis showed that, in the MNNG-induced rat model, Dendrobium polysaccharide markedly decreased the protein expression levels of Wnt-2β, β-catenin, and proliferating cell nuclear antigen (PCNA) while also altering the levels of endogenous serum metabolites (Zhao et al., 2019).

##### 4.2.1.3 Saponins

Ginsenoside Rg3 (GRg3) is one of the key metabolites of *Panax ginseng* C. A. Mey. After GRg3 treatment, gastric mucosal dysplasia in the Atp4a−/− mouse model had been significantly reduced (Xu et al., 2021a). Western blot analysis indicated that GRg3 targets the PI3K/AKT/mTOR signaling pathway to reduce the expression levels of PI3K, p-AKT, mTOR, and LDHA in cells from the GRg3-treated Atp4a−/− mouse model, thereby regulating glycolysis in GPL (Liu et al., 2020). In addition, studies have shown that GRg3 promotes apoptosis and inhibits abnormal cell proliferation by increasing the level of ROS production, thereby delaying the progression of GPL (Lv et al., 2022).

Panax notoginseng saponins have been mainly extracted from the traditional Chinese medicine *Panax notoginseng* (Burkill) F. H. Chen ex C. H. Chow. By inhibiting the PI3K/AKT/mTOR signaling pathway, Panax notoginseng saponins promoted autophagy in the MUN-induced GPL rat model. Western blot analysis showed that Panax notoginseng saponins inhibited the expression of PI3K/mTOR phosphorylation and p62 and increased the Beclin-1 and LC3 expression. Meanwhile, histological staining of the gastric mucosa in the rat model showed that Panax notoginseng saponins restored N-nitroso-N-methylurea-induced tissue disruption, increased mucin secretion in the gastric mucosa, protected the gastric mucosa, and ultimately reversed IM (Wang X. et al., 2024).

#### 4.2.2 Other signaling pathways

##### 4.2.2.1 Triterpenoids

*Celastrus orbiculatus* extract is the primary extract obtained from the stem of the traditional Chinese medicinal drug *Celastrus orbiculatus* Thunb. Studies have shown that treatment with *Celastrus orbiculatus* extract in the MNNG-induced PLGC rat model repaired gastric mucosal injury, restored cell morphology and tissue integrity, upregulated the expression of the tumor suppressor gene programmed cell death 4 (PDCD4), downregulated the expression of *ATG5*, suppressed the expression of the autophagy marker LC3-II, and upregulated the p62 protein level. This result suggests that Celastrus orbiculatus extracts inhibit autophagy levels by regulating the PDCD4–*ATG5* pathway and reversing PLGC. Furthermore, by reducing the expression of leucine-rich repeat-containing G-protein coupled receptor 5 (Lgr5), Celastrus orbiculatus extracts may suppress the EMT process in MGC-803 cells and contract PLGC, thereby influencing the expression of proteins associated with EMT (Zhu YD. et al., 2018; Wang et al., 2023).

#### 4.2.3 Summary and perspective on phytochemicals in GPLs

Phytochemicals show considerable promise in halting or reversing the progression of GPLs. The PI3K/AKT/mTOR signaling pathway emerges as a central hub, with multiple metabolites—including ginsenoside Rg3, astragaloside IV, and Panax notoginseng saponins—converging on this axis to restore autophagy and inhibit glycolysis. Additionally, other pathways such as Wnt/β-catenin and PDCD4–*ATG5* are implicated, suggesting a diverse mechanistic landscape.

Despite these findings, significant challenges persist. A major limitation is the predominant use of MNNG-induced rat models, which, while useful, may not fully recapitulate the complex etiology of human precancerous lesions, often driven by chronic inflammation and *H. pylori* infection. Furthermore, the majority of studies report only single-time-point assessments, failing to capture the dynamic interplay between autophagy and lesion progression.

### 4.3 Gastric cancer phase

During the gastric cancer phase, autophagy plays a dual role, acting as both a tumor suppressor and a pro-survival mechanism. Phytochemicals exert their anti-cancer effects by modulating autophagy through various signaling networks. The majority influence the PI3K/AKT/mTOR axis, while others engage the MAPK, AMPK, and nuclear factor kappa B (NF-κB) pathways to induce autophagic cell death or inhibit pro-tumoral autophagy. This section systematically categorizes these metabolites based on the signaling pathways they predominantly regulate.

#### 4.3.1 PI3K/AKT/mTOR signaling pathway

##### 4.3.1.1 Flavonols

Quercetin facilitated the transition of LC3-II and increased the generation of Beclin-1 and *ATG*-related gene expression. Moreover, further studies have shown that quercetin activates autophagy by inhibiting the phosphorylation of AKT, mTOR, and hypoxia-inducible factor 1 alpha (HIF-1α). An increased rate of quercetin-induced apoptosis was observed after treatment with the autophagy inhibitor chloroquine (CQ); at the same time, Bcl-2 protein expression levels were increased, whereas Beclin-1 protein expression levels were decreased. These results indicate that quercetin can stimulate protective autophagy in GC cells (Wang et al., 2011).

Isorhamnetin is a natural flavonoid product isolated from plants such as *Hippophae rhamnoides* Linn., *Ginkgo biloba* L., and *Tamarix ramosissima* Ledeb. In the CoCl_2_-induced hypoxic MKN-45 cellular model, isorhamnetin decreased PI3K/AKT/mTOR phosphorylation, reduced hypoxia-induced LC3-II accumulation, and downregulated p62 protein expression, thereby inhibiting autophagy in MKN-45 cells under hypoxic conditions. Furthermore, treatment with the autophagy inhibitor 3-methyladenine (3-MA) attenuated isorhamnetin-induced autophagy and increased apoptosis activity, as evidenced by the elevated expression of the apoptosis-related factors cleaved caspase-3 and cleaved caspase-9 expression, thereby promoting cell death (Li C. et al., 2021).

Myricetin is a naturally occurring flavonol metabolite originally isolated from the bark of *Morella rubra* Lour. Myricetin increased the protein expression levels of LC3-II and Beclin-1 while reducing the Bcl-2/Bax ratio and p-AKT expression level. When myricetin was combined with the autophagy inhibitor 3-MA, the viability of GC cells was reduced compared with treatment using myricetin alone. Meanwhile, in LY294002 (PI3K inhibitor)-pretreated AGS cells, myricetin reduced the Bcl-2/Bax ratio, accompanied by a reduction in cell viability. These results suggested that myricetin targets the PI3K/AKT/mTOR signaling pathway to promote autophagy in GC cells (Han et al., 2022).

##### 4.3.1.2 Stilbenoids

Dihydroceramide is a ceramide precursor synthesized from head sphingolipids. The dihydroceramide desaturases (Des1 and Des2) convert dihydroceramide to ceramide. A growing body of data suggests that dihydroceramide can regulate autophagy by modulating autophagy-associated factors, such as AKT, mTOR, JNK, and *ATG* proteins (Zheng et al., 2006; Alizadeh et al., 2023). In HGC-27 GC cells, resveratrol promoted autophagy through the inhibition of dihydroceramide desaturase activity, leading to the accumulation of dihydroceramide and increasing the production of the autophagic flux LC3-II (Signorelli et al., 2009). Resveratrol was also able to inhibit the phosphorylation of AKT, mTOR, and p70S6K through the activation of PENT, thereby reversing the EMT process in SGC-7901/DOX cells and increasing their sensitivity to DOX treatment (Xu et al., 2017).

##### 4.3.1.3 Polyphenols

It was shown that, in curcumin-treated AGS GC cells, the expression levels of PI3K, P-AKT, and P-mTOR were reduced, while the viability, migration, and invasion of GC cells were inhibited. In addition, in BGC-823 and SGC-7901 cells, curcumin had also been found to increase the formation of acidic vesicular organelles (AVOs), promote LC3 conversion, and result in the increased expression of autophagic proteins such as *ATG5* and *ATG7* (Xi et al., 2023). Further studies showed that after the use of an autophagy inhibitor, an increased rate of apoptosis was found in GC cells, suggesting that curcumin induces cytoprotective autophagy in GC cells (Fu et al., 2018; Li W. et al., 2017).

Ellagic acid is a naturally occurring polyphenolic metabolite. It suppresses the phosphorylation levels of the PI3K/AKT signaling pathway while simultaneously promoting LC3-II accumulation (Lu G. et al., 2023). Moreover, the metabolite urolithin A of ellagic acid regulated the PI3K/AKT/mTOR signaling pathway and induced autophagy to moderate the proliferation, migration, and invasion of HGC-27 and MKN-45 cells. *In vivo* experiments showed that in MKN-45-induced xenograft rat models, urolithin A reduced p-AKT expression and suppressed tumor growth. Further studies showed that combining urolithin A with the autophagy inhibitor 3-MA or CQ enhanced its tumor-suppressive effect, suggesting that urolithin A stimulates cytoprotective autophagy in GC cells (Zhang et al., 2023).

Proanthocyanidins are a natural product extracted from grape seeds. Western blot indicated that in MGC-803 cells, proanthocyanidins significantly inhibited the phosphorylation of PI3K, AKT, and mTOR in the PI3K/AKT/mTOR signaling pathway, promoted conversion of LC3-I to LC3-II, and increased the expression of caspase-9 and Beclin-1. Moreover, when 3-MA was added to proanthocyanidin-treated cells, nuclear damaged was observed, and the number of apoptotic vesicles increased. Quantitative fluorescence PCR analysis showed that 3-MA markedly inhibited the expression of Beclin-1 and Bcl-2, increased cell apoptosis, and enhanced the cytotoxicity of proanthocyanidins (Nie et al., 2016).

Proanthocyanidin B2 (PB2) is also a proanthocyanidin flavonoid polyphenolic metabolite found. The administration of PB2 to SGC-7901 cells inhibited the phosphorylation of AKT and mTOR while upregulating the expression of LC3, Beclin-1, and *ATG5*, thereby inducing cell autophagy and ultimately suppressing GC cell viability. The researchers also found that treatment with PB2-induced SGC-7901 cells with 3-MA increased cell viability, indicating that PB2 may induce GC cell apoptosis by targeting autophagy (Li Y. et al., 2021).

Corilagin is a polyphenolic tannic acid extracted from Euphorbiaceae and foliage plants. In the MNNG-induced GC rat model, corilagin suppressed the PI3K/AKT signaling pathway and the expression of Bcl-2, stimulate the activation of the apoptosis-related factors caspase-3 and caspase-9, and induced apoptosis in GC cells, ultimately leading to GC cell death (Zhang L. et al., 2022).

##### 4.3.1.4 Alkaloids

Berberine exhibits potent anti-inflammatory activity and also plays an important role in GC. BER has the ability to induce autophagy in BGC-823 cells both *in vitro* and *in vivo*. BER targets MAPK and inhibits the PI3K/AKT/mTOR/p70S6K signaling pathway while upregulating the formation of AVOs and the expression of Beclin-1 and LC3-II, thereby inducing BGC-823 cells’ autophagy (Zhang et al., 2020). In SGC-7901 cells, BER induces autophagy by regulating mTOR and MAPK signaling pathways (Yang et al., 2018). Additionally, research also showed that BER increased the therapeutic benefit of cisplatin (DQ) by suppressing the expression of the PI3K/AKT/mTOR signaling pathway in SGC-7901/DDP and BGC-823/DDP cell lines (Kou et al., 2020). According to the aforementioned findings, BER can inhibit the growth of GC cells by regulating their autophagy mechanism.

Tetrandrine is a natural bisbenzylisoquinoline alkaloid isolated from *Stephania tetrandra*. It was found that tetrandrine treatment of HGC-27 cells increased the formation of green fluorescent protein (GFP)-LC3 puncta and the levels of LC3-II. It not only suppresses the expression of p62, which is associated with autophagic flux but also does so in a concentration-dependent manner. Tetrandrine also decreased the phosphorylation of AKT/mTOR, PS6K, and P-4EBP1. Furthermore, treatment with the autophagy inhibitor 3-MA or bafilomycin A1 (Baf-A1) reduced the viability of tetrandrine-treated GC cells (Bai et al., 2018). The results above suggest that tetrandrine-induced autophagy may promote cancer cells’ survival. Furthermore, it has also been shown that tetrandrine induces ROS production in cells, which, in turn, promotes mitophagy (Wang H. et al., 2015).

Matrine is a natural metabolite from the roots and stems of the leguminous plant *Sophora flavescens* Ait. The administration of MA to SGC-7901 cells notably increased the number of AVOs present in the cytoplasm, and matrine increased Beclin-1 and LC3-II protein expression dose- and time-dependently. Matrine also increased the phosphorylation of AKT/mTOR but decreased total AKT and mTOR expression levels (Zhang et al., 2011; Li et al., 2013). It was also shown that the concentration of LC3-II, an autophagy marker, was increased in BGC-823 and AGS cells after treatment with matrine. Matrine inhibited autophagic degradation by impairing the function of lysosomal protease in SGC-7901 cells, thereby leading to the accumulation of LC3-II and AVOs (Wang et al., 2013).

##### 4.3.1.5 Flavonoids

Apigenin (APG) is a flavonoid metabolite that exists mainly in plants and fruits (Yan et al., 2017; Jang et al., 2022). Through the inhibition of the PI3K/AKT/mTOR signaling pathway, APG could trigger autophagy. It also elevated the expression levels of Beclin-1 and LC3-II and the production of AVOs dose-dependently, inhibited p62 protein expression in AGS cells, and changed the LC3-II in APG-exposed cells by administering the autophagy inhibitor CQ/3-MA. According to the aforementioned findings, APG could cause autophagy in GC cells (Kim et al., 2020). Certain studies have demonstrated that nobiletin treatment increases the expression levels of Beclin-1 and LC3-II. Nobiletin was also found to inhibit the phosphorylation of the AKT and the mTOR signaling pathway, decrease p62 protein expression, and regulate autophagy in GC cells. Treatment with the autophagy inhibitor CQ significantly reduced cell viability, suggesting that nobiletin may induce protective autophagy in GC cells (Moon and Cho, 2016).

Pectolinarigenin is a natural flavonoid in *Libanotis seseloides* with multiple pharmacological efficacies. Pectolinarigenin induced autophagy by decreasing the phosphorylation of the AGS and MKN-28 cells’ PI3K/AKT/mTOR signaling pathway. It also promoted the accumulation of AVOs dose-dependently, increased the LC3-II level, promoted the formation of autophagosomes, downregulated Beclin-1 expression, and activated and cleaved poly(ADP-ribose) polymerase (PARP) to induce GC cell death (Lee et al., 2018). Pectolinarigenin can also induce GC cell mortality in the AGS xenotransplantation mouse model by controlling the PI3K/AKT/mTOR, HIF-1, and Wnt pathways (Lee et al., 2024).

*Polygoni orientalis* Fructus is a flavonoid extracted from the traditional Chinese medicinal plant *Reynoutria japonica* Houtt. According to studies, eriodictyol is the most potent anti-GC flavonoid in Polygoni orientalis Fructus. Eriodictyol blocked the PI3K/AKT pathway, reduced the phosphorylation of Ser473 of AKT, and decreased PI3K phosphorylation in GC cells dose-dependently while inhibiting AGC, MKN-45, and HGC-27 cell proliferation. Furthermore, apoptosis in GC cells is induced by increased cleavage of the apoptotic protein PARP-1 and accumulation of caspase-3 in GC cells treated with eriodictyol (Shan et al., 2022).

Cynaroside is a flavonoid glycoside metabolite extracted from the traditional Chinese medicinal drug honeysuckle, which has pharmacological effects. Research has demonstrated that the mesenchymal–epithelial transition (MET) protein played a role during the initial phase of the AKT/mTOR signaling pathway (Xu et al., 2022). In MKN-45 and SGC-7901 cells, cynaroside inhibited cell growth, invasion, and migration. The ubiquitination assay showed that cymaroside enhanced the ubiquitination of MET and decreased the phosphorylation of AKT/mTOR/P70S6K, which may be associated with its anti-tumor effect (Ji et al., 2021).

##### 4.3.1.6 Organosulfur compounds

Allicin is a natural organosulfur product in *Allium sativum* L. Allicin induced autophagy in MGC-803, BGC-823, and SGC-7901 cell lines by modulating p38 signaling and downregulating phosphorylation of the PI3K and AKT pathways in GC cells (Sun and Wang, 2003). Meanwhile, allicin could also induce SGC-7901 cell apoptosis dose-dependently, increase ROS production within the mitochondria, and block the G2/M phase of the cell cycle (Lv et al., 2020).

##### 4.3.1.7 Lignans

Magnolol was extracted from *Houpoea officinalis* cortex. Magnolol induces autophagy in SGC-7901 cells by inhibiting the PI3K/AKT signaling pathway and dose-dependently enhancing autophagic vacuole formation, thereby protecting cells from damage. Further studies indicated that treatment with 3-MA increased cell viability and the expression levels of Bcl-2 and Bax proteins (Rasul et al., 2012). Additionally, studies showed that magnolol impairs mitochondrial function, triggers the PTEN-induced kinase 1 (PINK1)-pUb-PRKN positive feedback loop, and induces autophagy in the LC3-OPTN-CALCOCO2/NDP52 feedback loop (Tang et al., 2021).

##### 4.3.1.8 Xanthones

Mangiferin, a naturally occurring polyphenolic metabolite, is most abundant in mangoes and other Mangifera L. plants of the family Anacardiaceae. Mangiferin targets the PI3K/AKT/mTOR signaling pathway to promote autophagy in SGC-7901 and BGC-823 cells, thereby inhibiting GC progression (Du et al., 2018). In addition to that, studies have also found that mangiferin stimulates autophagy by decreasing p62 expression, dose-dependently increasing LC3-II conversion, enhancing autophagic flux, and inducing autophagy (Hou et al., 2018).

##### 4.3.1.9 Triterpenoids

Cucurbitacin B is a triterpenoid extracted from the Cucurbitaceae Juss. plants. Research has shown that it can induce autophagy. In SGC-7901/DDP cells that have been treated with cucurbitacin B, it inhibited GC cells by inhibiting the cancerous inhibitor of protein phosphatase 2A (CIP2A)/protein phosphatase 2A (PP2A)/mTORC1 signaling axis, upregulating LC3-II and Beclin-1 expression, leading to an increased susceptibility of DDP, and inhibiting GC cell proliferation (Liu et al., 2017).

##### 4.3.1.10 Chalcones

Flavokawain B (FKB) is a naturally occurring chalcone identified in extracts derived from the roots of *Piper methysticum* G. Forst., which is abundant in fruits, vegetables, and edible plants. In the AGS-transplanted nude mouse model, FKB targeted the mTOR signaling pathway and increased ROS accumulation and the expression levels of autophagy factors LC3-II, Beclin-1, and *ATG7* dose- and time-dependently to inhibit the colonization ability of AGS cells. Further studies found that although FKB does not induce apoptosis in AGS cells, Western blot analysis showed that as Bcl-2 expression decreased, Beclin-1 expression increased and was associated with the AGS cell death (Chang et al., 2017). There is also experimental evidence that FKB induces cellular autophagy by activating the activating transcription factor (AFT)–DDIT3 signaling pathway, thereby inhibiting the AKT/mTOR signaling pathway (Wang et al., 2018).

##### 4.3.1.11 Bufadienolides

Bufalin is extracted from the toad skin, a traditional Chinese medicine known for its anti-cancer properties. Research has shown that bufalin suppressed the phosphorylation of the AKT/mTOR/p70S6K signaling pathway and dose- and time-dependently increased the LC3-II and Beclin-1 protein levels; the number of autophagic vesicles increased, while p62 protein expression decreased. The expression of *ATG5–ATG12* conjugate protein and the *ATG9–*autophagy-related 16-like 1 complex initially increased and then decreased. Meanwhile, Western blot analysis revealed that it also increased the phosphorylation level of ERK1/2 and the expression of ER stress proteins, inositol-requiring enzyme 1 (IRE1), C/EBP homologous protein (CHOP), and phospho-eukaryotic initiation factor alpha (eIFa). Further studies showed that, following combined treatment with bufalin and CQ, instrument analysis demonstrated an increase in cleaved PARP levels and the number of apoptotic cells. Overall, bufalin induces autophagy in GC cells by inhibiting the PI3K/AKT/p70S6K pathway and activating the ERK pathway; bufalin can provide both anti-tumor and apoptosis protection (Qi et al., 2019). In addition, combining 3-MA or *ATG5* siRNA with bufalin revealed that the level of apoptotic factors such as caspase-3 and -8 was increased, which promoted cell death. The above results suggest that bufalin-induced activation of autophagy through ER stress is cytoprotective (Zhao H. et al., 2017).

##### 4.3.1.12 Diterpenoids

Tanshinone is an abietane diterpene extracted from *Salvia miltiorrhiza* Bunge. Some studies have shown that although BCL-2 is an apoptosis-related protein that can regulate apoptosis, it can also participate in autophagy. In addition, Bcl-2 inhibits autophagy by conditioning the PI3K/AKT pathway and activating mTOR. In tanshinone I-induced BGC-823 and SGC-7901 cells, Western blot analysis detected that tanshinone I increased the generation of LC3-II, increased the cleavage level of caspase 3 and PARP, increased Bax expression, and inhibited the expression of the autophagy negative regulator Bcl-2, which, in turn, prevented the formation of complexes of Beclin-1 and Bcl-2. Moreover, immunoprecipitation results showed that Bcl-2 overexpression increased the Bcl-2/Beclin-1 ratio, ultimately leading to a reduction of the vacuolar protein sorting 34 (VPS34)/Beclin-1 complex formation and inhibiting autophagosome generation (Zhou et al., 2011). *In vitro* and *in vivo* experiments combining tanshinone I and CQ treatment in BGC-823 tumor xenografts in rats, as well as in BGC-823, and SGC-7901 cells, showed that this combination inhibited LC3-II conversion, increased the expression levels of Bcl-2 and caspase-3, and reduced tumor volume in rats. The results suggested that tanshinone I caused cytoprotective autophagy and promoted gastric tumor death (Jing et al., 2016). Tanshinone IIA can promote cellular autophagy, decrease the amount of p62 protein, increase LC3B-II expression levels, and enhance the therapeutic effect of DOX in SNU-719, SNU-719R, and SNU-620 GC cells when combined with doxorubicin (Xu et al., 2018).

Paclitaxel is a major anti-cancer drug found in the bark of the *Taxus wallichiana* var. *chinensis(Pilg.) Florin*, which is effective in treating stomach cancer (Galletti et al., 2020). Paclitaxel treatment was found to increase the formation of AVOs in AGS and BGC-823 cells, reduce the expression levels of mTOR and p62, and increase the expression levels of LC3B-II, sequestosome 1, PI3K II, *ATG* proteins, and Beclin-1 proteins in a time-dependent manner (Khing et al., 2021; Yu et al., 2017; Cheng et al., 2022).

Oridonin is a natural tetracyclic diterpenoid isolated from *Rabdosia* (Blume) Hassk. Oridonin has been demonstrated to dose-dependently enhance the induction of the autophagy marker LC3B in HGC27, MGC803, and AGS cells (Liu et al., 2012; Zhou et al., 2007). Additionally, treatment of oridonin-induced cells with BafA1 resulting in decreased PARP and caspase-3 cleavage levels, thereby promoting apoptosis (Wang Y. et al., 2021). According to the studies mentioned above, oridonin can induce autophagy and subsequently promote apoptosis, thereby increasing cancer cell death.

Cordyceps militaris produces a novel diterpene molecule called Jaridon 6. When Jaridon 6 was applied to MGC-803 GC cells, it was found that the cells exhibited increased expression of the autophagy marker LC3-II and decreased p62 protein expression. Additionally, Jaridon 6 concentration-dependently inhibited the PI3K/AKT pathway, thereby inhibiting the sirtuin 1 (SIRT1) enzyme activity and inducing autophagy (Fu et al., 2021).

##### 4.3.1.13 Carotenoids

Fucoxanthin is one of the major carotenoids present in seaweeds. Research has shown that fucoxanthin upregulated the expression of Beclin-1 and LC3, increasing autophagosome accumulation (Lee et al., 2021). After co-treatment of SGC-7901 cells with 3-MA and fucoxanthin, the upregulating effect of fucoxanthin was reduced, whereas Bcl-2 expression increased (Zhu Y. et al., 2018), indicating that fucoxanthin-induced autophagy influences GC cell apoptosis and reduces cell viability. Fucoxanthin can also control autophagy by inhibiting the mTOR signaling pathway, thereby reducing the phosphorylation of the AKT, p53, and p70S6K, while also reducing oxidative stress and mitochondrial damage (Ahmed et al., 2022; Hou et al., 2013).

Astaxanthin is a natural fat-soluble lutein-like carotenoid. In gastric epithelial AGS cells infected by *H. pylori*, cell death increased, as has the release of caspase-3. *In vivo* experiments showed that it decreased the phosphorylation of AKT and mTOR while increasing the phosphorylation of AMPK and the expression of LC3-II and p-*ULK1* in *H. pylori*-induced AGS cells, thereby promoting autophagy and effectively inhibiting apoptosis (Lee et al., 2020). In addition, astaxanthin inhibits MMP-7 and MMP-10 expression by suppressing the activity of the PI3K/AKT/mTOR/NF-kB signaling pathway in the cells infected with *H. pylori*. According to the above results, astaxanthin may prevent *H. pylori*-associated gastric cancer by inhibiting the growth, migration, and invasion of gastric cancer cells (Lee et al., 2022).

##### 4.3.1.14 Steroidal saponins

Polyphyllin I is a steroidal saponin derived from the plant *Paris polyphylla* Smith. Research showed that the treatment of polyphyllin I in HGC-27 cells dose-dependently inhibited the phosphorylation of the PI3K/AKT/mTOR signaling pathway, increased the conversion of LC3-II, and downregulated p62 protein expression and its pathway’s downstream effectors, ultimately leading to HGC-27 cells’ autophagy and preventing the growth of GC (He et al., 2019).

(25R)-5α-spirostan-3β-yl-3-O-acetyl-O-β-d-glucopyranosyl-(1→2)-O-[β-d-glucopyranosyl-(1→3)]-O-β-d-glucopyranosyl-(1→4)-β-d-galactopyranoside (A-24) is a steroidal saponin extracted from *Allium* L. species. A-24 has been indicated to inhibit Beclin-1 and LC3-II expression and reduce the expression levels of the p62 protein while enhancing the occurrence of autophagy in SGC-7901 and AGS cells. Treatment with autophagy inhibitors suppressed A-24-induced autophagic activity and increased the expression of pro-apoptotic factors, such as Bax and caspase-3, in two types of A-24-induced GC cells, demonstrating that A-24-induced autophagy was cytoprotective (Xu et al., 2020). Additionally, A-24 increased ROS production in *p53* wild-type or p53-deficient AGS cells and mediated autophagy through the ROS, PI3K, AKT, and mTOR pathways, thereby inhibiting GC metastasis and promoting cell death (Xu et al., 2021b).

##### 4.3.1.15 Monoterpenoids

Thymoquinone is a natural monoterpenoid extracted from *Nigella sativa* L. Thymoquinone was found to inhibit PI3K/AKT/mTOR phosphorylation, increase autophagosome formation, upregulate LC3-II expression while simultaneously reducing p62 levels in GC cells, and promote autophagy. The results indicated that the treatment with the autophagy inhibitor BafA1 reduced thymoquinone-induced autophagy, while increasing the activity of apoptotic factors and further inhibiting GC cell viability. The above results indicated that thymoquinone induced protective autophagy in HGC-27 and MKN-45 cells (He et al., 2023). In addition, thymoquinone could inhibit the expression of the PI3K/AKT/mTOR signaling pathway in SGC-7901 and MGC-803 cells, upregulate E-cadherin, decrease N-cadherin, vimentin, and TWIST gene expression, and inhibit EMT in GC (Feng et al., 2017).

##### 4.3.1.16 Aldehydes

Cinnamaldehyde is a naturally occurring metabolite that is derived from *Cinnamomum cassia*. The cinnamaldehyde treatment of SNU-638, SNU-216, and other GC cell lines resulted in the suppression of p62 expression and an elevation in *ATG5*, Beclin-1, and LC3-II. In addition, cinnamaldehyde inhibited the combination of Bcl-2 to Beclin-1 and increased AMPK/*ULK1* phosphorylation while simultaneously inhibiting mTOR phosphorylation and promoting autophagy. After co-treatment with autophagy inhibitors, the cell activity decreased, indicating that cinnamaldehyde induced protective autophagy in GC cells (Kim, 2022).

##### 4.3.1.17 Flavanones

Naringin is a natural glycoside extracted from grapefruit. In naringin-treated AGS cells, naringin enhanced the production of autophagosomes, LC3-II, and Beclin-1 while also activating p-ERK1/2, p-p38, and p-JNK in the cells. Additionally, naringin activated the MAPK pathway, suppressed PI3K/AKT/mTOR phosphorylation, induced autophagy, and inhibited cancer cell growth (Raha et al., 2015; Raha et al., 2020). It has also been shown that naringin induced autophagy in SUN-1 cells by downregulating the expression of the PI3K/AKT signaling pathway. Researchers also found that it increased the expression of caspase-3 and Bax to induce cell apoptosis. In naringin-treated SUN-1 cells, pre-treatment with 3-MA reduced the expression of P-PI3K/PI3K and P-AKT/AKT, increased p62 expression, and attenuated the naringin-induced upregulation of LC3B-II, Beclin-1, and caspase-3. These results indicate that naringin can induces autophagy and apoptosis in GC cells, thereby inhibiting the development of GC (Xu C. et al., 2021).

##### 4.3.1.18 Chalcones

Isoliquiritigenin-17 is one of the analogs of isoliquiritigenin. When treating human SGC-7901 cells with isoliquiritigenin-17, it was found that the activities of LC3-II and Beclin-1 proteins in the cells increased, while the expression of p62 was suppressed. It was also found that isoliquiritigenin-17 inhibited the phosphorylation of the AKT and mTOR signaling pathway in human SGC-7901 cells. The above results suggest that isoliquiritigenin-17 may exert anti-cancer activity by attenuating the AKT/mTOR signaling pathway in human GC cells (Huang et al., 2020).

##### 4.3.1.19 Alkaloids

Magnoflorine is an alkaloid extracted from the rhizome of *C. chinensis* Franch. In SGC-7901, BGC-823, and MGC-803 cell lines, magnoflorine reduced cell proliferation. Treatment with magnoflorine dose-dependently downregulated AKT/mTOR and LAMP1 phosphorylation, suggesting that it induced autophagosome formation. In conclusion, magnoflorine targets the AKT/mTOR signaling pathway to inhibit GC growth (Sun XL. et al., 2020).

##### 4.3.1.20 Glycosides

Salidroside is a natural metabolite extracted from the traditional Chinese medicinal plant *Rhodiola rosea* Linn. The results indicated that in AGS cells, treatment with salidroside reduced the phosphorylation of PI3K, AKT, and mTOR while increasing the expression levels of LC3-II and Beclin-1 in a concentration-dependent manner. Furthermore, treatment with the autophagy inhibitor CQ results in a notable reduction in cellular activity, suggesting that salidroside promotes cytoprotective autophagy in AGS GC cell lines (Rong et al., 2020).

##### 4.3.1.21 Phenolic acids

Syringic acid is a phenolic product found in plants and fruits such as grains, olives, spices, squash, and grapes. Research has shown that syringic acid intervention in AGS cells suppressed cell viability, altered cell morphology, induced ROS expression, inhibited the phosphorylation of the AKT/mTOR signaling pathway, and suppressed the expression of inflammatory factors, such as IL-6, NF-κB, TNF-α, and COX-2. Syringic acid decreases the proliferation of AGS cells by inhibiting the AKT/mTOR signaling pathway (Pei et al., 2021).

##### 4.3.1.22 Nanomaterials

*Kaempferia parviflora* (black ginger) root extract was used to reduce selenium cations to form Kaempferia parviflora-derived selenium nanoparticles (KP-SeNPs). In AGS cells, KP-SeNPs were shown to increase autophagic flux. KP-SeNP treatment suppressed the PI3K/AKT/mTOR signaling pathway, reduced the p62 protein expression levels and the downstream effector of p70S6K, and increased the expression levels of LC3-II. Meanwhile, researchers also combined the KP-SeNPs alongside 740Y-P (activator of the PI3K pathway), which led to a reduction of PI3K, AKT, and 70S6K phosphorylation levels; upregulated the Bcl-2 expression and downregulated Bax and caspase 3 expressions; promoted AGS cell apoptosis; and induced tumor death. In conclusion, KP-SeNPs have been shown to induce apoptosis in AGS GC cells by promoting autophagy (Wang et al., 2022).

##### 4.3.1.23 Sesquiterpenoids

A naturally occurring metabolite derived from Curcuma longa Linn. is called β-elemene. β-elemene caused autophagy in MGC-803 and SGC-7901 cells *via* the PI3K/AKT/mTOR/P70S6K1 signaling pathway. It was shown that β-elemene could markedly induce the formation of autophagic vesicles, increase the expression of LC3-II and the *Atg5–Atg12* conjugated protein, and inhibit the viability of GC cells. In addition, after co-treatment with CQ/3-MA, viability and clonogenic ability decreased, and flow cytometry analysis showed that β-elemene increased the number of apoptotic cells, indicating that β-elemene could induce protective autophagy to prevent apoptosis-induced cell death (Liu et al., 2011).

#### 4.3.2 MAPK signaling pathway

##### 4.3.2.1 Flavonolignans

Silibinin is a polyphenolic flavonoid extracted from S*ilybum marianum.* The study observed that silibinin inhibited the invasive and migratory capacities of SGC-7901 cells. Further studies demonstrated that silibinin dose-dependently inhibited the expression of p-p38 MAPK, along with reducing the expression of MMP-2 and MMP-9, ultimately suppressing the activity of SGC-7901 cells (Lu et al., 2017). Moreover, it has been found that silibinin can inhibit TNF-α-induced MMP-9 expression by suppressing the activity of the MEK/ERK signaling pathway, thereby preventing GC cell metastasis (Kim et al., 2009).

##### 4.3.2.2 Flavonols

In isorhamnetin-treated HGC-27 GC cells, it was found that isorhamnetin dose-dependently inhibited the MAPK/mTOR signaling pathway, significantly increased E-cadherin and reduced Vimentin protein mRNA levels, and reversed EMT in GC cells (Shi et al., 2023).

##### 4.3.2.3 Stilbenoids

It was also found that Res could suppress tumor growth. In Res-treated SGC-7901 cell lines, resveratrol decreased the levels of phosphorylated AKT and mTOR expression, increased p38 MAPK and ERK phosphorylation levels, upregulated ER stress-related protein expression, including CHOP, decreased the levels of MMP2 and MMP9, activated autophagy and ER stress, dose-dependently upregulated the levels of autophagy-associated proteins p62 and LC3-II and the expression of mitosis-associated proteins, decreased the amount of Beclin-1 protein, and inhibited GC cell activity (Li et al., 2022).

##### 4.3.2.4 Flavonoids

Diosmetin is a natural flavonoid metabolite extracted from *Citrus reticulata Blanco* fruits. Diosmetin dose-dependently increased ERK1/2, p38, and JNK phosphorylation, activated the MAPK/JNK pathway, induced autophagy, enhanced the expression of microtubule-associated protein 1A/1B-light chain 3 beta (LC3B), and inhibited p62 expression in HGC-27 cells. Elevated cleaved caspase-3 and PARP were observed in HGC-27 cells pretreated with diosmetin in conjunction with an autophagy inhibitor or a JNK inhibitor. These findings indicate that diosmetin may facilitate protective autophagy in GC cells, and suppression of diosmetin-mediated autophagy can effectively inhibit cancer cell activity (Pan et al., 2023).

##### 4.3.2.5 Naphthoquinones

Plumbagin, a naturally occurring naphthoquinone metabolite, is isolated from two white-flowering plants of the Plumbago genus, Plumbago zeylanica (Ceylon leadwort) and Plumbago europaea (European leadwort). It was demonstrated that plumbagin enhanced autophagosome formation and increased the expression of autophagy-related markers, including Beclin-1, LC3-II, *ATG5*, and *ATG7*. It also inhibited the phosphorylation of AKT, mTOR, and PI3K (Pan et al., 2015; Zhang et al., 2021; Li et al., 2014; Lin et al., 2019; Kuo et al., 2006). Plumbagin can also induce autophagy by inhibiting the MAPK signaling pathway (Su et al., 2022; Wang F. et al., 2015).

##### 4.3.2.6 Saponins

Ginsenoside Rg5 (G-Rg5) is derived from the plant *Panax ginseng* C. A. Mey. In the BGC-823 and SGC-7901 cell lines, G-Rg5 enhanced LC3B-II, Beclin1, *ATG5*, and *ATG12* expression levels while concurrently decreasing the protein expression of P62 in a time- and concentration-dependent manner. Moreover, co-treating GC cells with 3-MA and G-Rg5 revealed that the expression of autophagy and apoptosis-related factors, including caspase-3, PARP, LC3B-II, and others, was inhibited, indicating that G-Rg5-mediated autophagy contributes to apoptosis and reduces GC cell viability. Rg5 also increased ROS production in the cells, activated the MAPK (p38, JNK, and ERK) pathway, and induced cell autophagy (Liu and Fan, 2019).

##### 4.3.2.7 Phenolics

The scarlet resin of Dracaena draco is the source of a ethyl acetate extract derived from the resin of the Chinese dragon’s blood. In HGC-27 and MGC-803 cells, research has demonstrated that Chinese dragon’s blood ethyl acetate extract significantly inhibited cell proliferation and migration capability. Further studies indicated that reduced mTOR and p62 expression levels and increased *ATG3*, *ATG5*, and LC3-II expression levels were observed in CDBBE-treated MGC-803 and HGC-27 cells, thereby promoting autophagic cell death (Ouyang et al., 2023).

#### 4.3.3 AMPK signaling pathway

##### 4.3.3.1 Flavonoids

In AGS and SNU-638 cell lines, APG enhanced the activity of AMPK and *ULK1* and inhibited mTOR phosphorylation. The expression levels of *ATG5*, Beclin-1, and LC3-II were detected by fluorescence quantitative RT-PCR and Western blot, which revealed a reduction in the p62 level. Additionally, it decreased the level of p62 by stimulating the activity of IRE1α, JNK, p-protein kinase R-like ER kinase (PERK), CHOP, and other proteins; it also induced ER stress, promoted autophagy in AGS and SNU-638 cells, and inhibited the growth of GC cells (Kim and Lee, 2021).

##### 4.3.3.2 Monoterpenoids

Perillaldehyde is a natural monoterpene metabolite derived from *Perilla frutescens*. The results of monodansylcadaverine (MDC) staining showed that the phosphorylation of AMPK at Thr172f was increased. Researchers also found that perillaldehyde promoted LC3-II expression, autophagosome formation, AMPK activation and phosphorylation, and Beclin-1 expression, thereby inducing autophagy and inhibiting GC cell growth in GC9811-P and MFC cells. Further research indicated that, after treatment of the xenograft GC mouse model with 3-MA and an AMPK inhibitor, the inhibitory effect of perillaldehyde on GC was reduced. Overall, perillaldehyde can activate AMPK and induce autophagy to inhibit GC cell growth (Zhang et al., 2019).

##### 4.3.3.3 Triterpenoids

Celastrol is a quinuclidinium methyl triterpenoid, the primary bioactive compound derived from the roots of the traditional Chinese medicine *Tripterygium wilfordii* Hook. f. In AGS and YCC cells, celastrol inhibited cell proliferation and migration, activated the AMPK pathway to inhibit the AKT/mTOR signaling pathway, and increased the expression of LC3, *ATG5*, *ATG7*, and Beclin-1 to induce autophagy. Further studies showed that celastrol increased the expression of proteins for cell cycle and apoptosis, and it also increased the amount of phosphorylated AMPK. Celastrol treatment also increased the expression of cytochrome c, caspase-3, -8, and -9, and Bax and reduced the expression of the anti-apoptotic proteins Bcl-2 and Bcl-2-like 1(Bcl-xl). These studies indicated that celastrol targets AMPK and blocks the AKT/mTOR signaling pathway to inhibit GC cell growth (Lee et al., 2014).

##### 4.3.3.4 Alkaloids

Harmine, a natural product isolated from the medicinal plant *Peganum harmala* L. In the MGC-803 and SGC-7901 cell lines, harmine blocked the PI3K/AKT/mTOR pathway, enhanced the expression of LC3-II, and elevated the levels of Beclin-1 protein, activated the AMPK signaling pathway, and increased phosphorylation of *ULK1*, ultimately resulting in the activation of autophagy. The co-treatment of harmine with the autophagy inhibitor 3-MA resulted in increased levels of cleaved-PARP and cleaved-caspase-3 and decreased cancer cell viability, indicating that harmine-induced autophagy may contribute to the induction of apoptosis in cancer cells (Li C. et al., 2017).

##### 4.3.3.5 Flavonols

Kaempferol is a natural flavonoid metabolite extracted from the roots of *Kaempferia galanga* Linn, which has anti-inflammatory, antioxidant, anticancer, and gastroprotective properties. With kaempferol treatment following the knockdown of the AMPK and *ULK1* genes in GC cells, kaempferol-induced elevation of LC3-II levels was inhibited, and it was found that blocking AMPK-α expression prevented kaempferol-mediated autophagic cell death (Campos-Vidal et al., 2021). An *in vitro* experiment compared the viability inhibitory effects of kaempferol with thapsigargin (an ER stress inducer) on GC cells, revealing that kaempferol was able to bind to the inositol-requiring 1 (IRE1)/JNK/CCAAT enhancer *via* the CHOP, AMPK/*ULK1*, and histone deacetylase (HDAC)/G9a (histone lysine 9 dimethylation-specific methyltransferase) pathways, which caused autophagic cell death in AGS, SNU-216, and other cell lines and reduced the survival of GC cells (Kim et al., 2018; Li et al., 2018).

##### 4.3.3.6 Indoles

3,3′-Diindolylmethane is a naturally occurring metabolite extracted from cruciferous vegetables such as cauliflower. In both *in vivo* and *ex vivo* experiments, 3,3′-diindolylmethane exhibited cancer cytotoxicity and dose-dependently enhanced LC3-II and *ATG5* expression in BGC-823 and SGC-7901 cell lines. In addition, when BGC-823 cells with 3-MA and BafA1 were used, PI3K activity was inhibited, but LC3-II accumulation was not affected. Further studies indicated that 3,3′-diindolylmethane regulated *ATG5* expression in GC cells by inhibiting miR-30e expression, thereby promoting LC3-II degradation, restoring lysosomal function, promoting autophagy, and inhibiting GC cell viability (Ye et al., 2016). In addition, it was shown that 3,3′-diindolylmethane, by upregulating the expression of AMPK/stromal interaction molecule 1 (STIM1)/ATF6 (activating transcription factor 6), concentration-dependently increases CHOP expression to promote ER stress and elevates the expression levels of LC3-II, Bcl-2, and caspase-3, as well as triggers autophagy and apoptosis in GC cells to inhibit GC (Ye et al., 2021).

#### 4.3.4 NF-κB signaling pathway

##### 4.3.4.1 Phenylpropanoids

Eugenol is the major active metabolite of *Syringa oblata* Lindl. It was shown that, in the MNNG-induced GC cell model, eugenol suppressed NF-kB expression, upregulated p21 and *p53* protein expression levels and inhibited MNNG-induced GC in rats (Manikandan et al., 2011). Additionally, research showed that eugenol could induce autophagy by regulating the expression of autophagy-related proteins, such as p62, LC3-II, and p21 (Abdullah et al., 2021; Sun X. et al., 2020).

##### 4.3.4.2 Proteins

Trichosanthin is a plant metabolite extracted from the rhizomes of *Trichosanthes* L. Trichosanthin increased ROS production in MKN-45 cells dose-dependently, promoted p-NF-kB and *p-p53* expression levels, and consequently induced the expression of autophagy-associated factors LC3-II and *ATG5*, induced autophagy in MKN-45 cells, and inhibited tumor growth (Wei et al., 2016).

#### 4.3.5 Apoptosis- and autophagy-related pathways

##### 4.3.5.1 Triterpenoids

Betulinic acid is a pentacyclic triterpenoid isolated from *Betula, Platanus* L., and *Eucalyptus* LHér. In SGC-7901 cells, betulinic acid inhibited p62 expression and upregulated Beclin-1 expression, leading to autophagosome formation, and it also reduced the phosphorylation of ERK and MAPK/ERK kinase (MEK), thereby inhibiting GC cell growth. Further research showed that pretreatment with 3-MA in SGC-7901 and BGC-823 cells reduced the expression of cleaved caspase-3, cleaved PARP, and LC3-II, inhibited apoptosis, and restored cell viability (Wang H. et al., 2021).

##### 4.3.5.2 Chalcones

Isoliquiritigenin is a flavonoid extracted from *G. uralensis* Fisch. root with anti-cancer bioactivity. In isoliquiritigenin-treated MKN-28 cells, the cell proliferation was reduced, and the LCE-II/LC3-I ratio was increased. Beclin-1 expression was also increased, whereas p62 expression was decreased, indicating that isoliquiritigenin triggered autophagy in MKN-28 cells (Zhang XR. et al., 2018).

Alpha, 2′-dihydroxy-4,4′-dimethoxydihydrochalcone is a citrulline metabolite isolated mainly from the Meliaceae Juss. family. It was shown that alpha, 2′-dihydroxy-4,4′-dimethoxydihydrochalcone induced dose-dependent increases in the expression levels of *ATG5*, *ATG7*, and Beclin-1 and promoted the conversion of LC3-I to LC3-II in MKN-45 cells, thereby increasing autophagosome formation. It also impeded the proliferation, invasion, and migration of MKN-45 cells and inhibited GC cell growth(Wan et al., 2018).

##### 4.3.5.3 Alkaloids

Solanine is a naturally occurring steroidal glycoalkaloid in the *Solanum* (Section Solanum) with anti-cancer and other activities. It has been shown that activation of the MYC proto-oncogene induces ER stress-mediated autophagy, and AAMDC (adipogenesis-associated Mth938 domain-containing) activates the PI3K/AKT/mTOR signaling pathway (Dey et al., 2013). It also regulates the translation of AFT4 and MYC. In GES-1, MGC-803, and SGC-7901 cell lines, solanine reduced gastric tumorigenesis (Golden et al., 2021). Solanine treatment elevated the ratio of LC3-II to LC3-I and reduced the overexpression of adipogenesis-associated Mth938 domain-containing, MYC, ATF4, and Sesn2 proteins. It also enhanced the concentrations of caspase-3 and Bax and downregulated Bcl-2 expression. Solanine exerts inhibitory effects on GES-1, both *in vitro* and *in vivo*, by suppressing adipogenesis-associated Mth938 domain-containing protein-mediated autophagy inactivation (Tang et al., 2023).

##### 4.3.5.4 Polyphenols

After corilagin treatment, cellular morphology was altered, including a reduced cell number and a rounded morphology, and ROS accumulation was induced. Western blot analysis and AO dye showed that corilagin dramatically increased the expression levels of the autophagy biomarker LC3-II and induced AVO formation in SGC-7901 and BGC-823 cell lines (Xu J. et al., 2019).

##### 4.3.5.5 Stilbenoids

Piceatannol is a naturally occurring stilbene metabolite. Treatment of GC cell lines, such as BGC-823 and MKN-28, with piceatannol increased LC3-II expression levels in cancer cells. In addition, piceatannol binds to Beclin-1, blocks the interaction between Bcl-2 and Beclin-1 and Beclin-1 phosphorylation at the Ser-295 site, promotes cellular autophagy, and inhibits GC development (Huangfu et al., 2023).

Pterostilbene is a naturally occurring stilbene metabolite found predominantly in grapes and exhibits biological activities similar to those of resveratrol.^265^ In N-87 and MKN-45 gastric cell lines, pterostilbene in combination with sorafenib promoted the formation of AVOs, increased LC3-II expression, inhibited p62 protein expression, promoted autophagy, and inhibited tumor activity (Zhao et al., 2020). In addition, pterostilbene can induce autophagy by downregulating p62 expression, increasing the expression levels of Beclin-1, *ATG5*, and microtubule-associated protein LC3-II, activating JNK1/2, and inhibiting the activation of AKT, ERK1/2, and p38 (Lin et al., 2020).

##### 4.3.5.6 Sesquiterpenoids

A naturally occurring chemical called dihydroartemisinin (DHA), which has anti-malarial and anti-cancer properties, was identified from *Artemisia carvifolia* and its derivatives. Research has indicated that DHA enhances LC3-II expression, promotes the degradation of the autophagy substrate p62, and increases the formation of GFP-LC3-II puncta (Che et al., 2020). DHA also increased the E-cadherin level while suppressing the AKT, p-AKT, and Snail proteins. These effects were observed in a time- and dose-dependent manner, along with the inhibition of the PI3K/AKT pathway and the regulation of EMT-related proteins. Consequently, DHA reduced GC progression and reversed the EMT process in SGC-7901 cells (Li et al., 2019). Additionally, DHA enhanced SGC-7901/DDP sensitivity to DDP, which, when combined with DQ, showed a notable anti-cancer activity (Zhang S. et al., 2022). The results above suggested that DHA regulates PI3K/AKT, LC3-II, p62, and other components to mediate autophagy. Additionally, DHA was shown to promote cancer cell death by increasing ERK1/2, JNK1/2, and p38 MAPK phosphorylation levels in BGC-823 cells (Zhang et al., 2017).

#### 4.3.6 Summary and perspective on phytochemicals in gastric cancer

The investigation of phytochemicals in gastric cancer reveals a complex landscape where autophagy serves as a double-edged sword. A predominant finding is that many phytochemicals, including curcumin, quercetin, and berberine, induce cytoprotective autophagy by inhibiting the PI3K/AKT/mTOR pathway. This protective mechanism can paradoxically limit the efficacy of these agents, as evidenced by increased cancer cell death upon co-treatment with autophagy inhibitors (3-MA and CQ). This observation highlights a crucial therapeutic implication: the combination of phytochemicals with autophagy inhibitors could represent a rational strategy to enhance their anti-cancer efficacy.

Conversely, other agents such as tanshinone I, bufalin, and thymoquinone appear to induce autophagic cell death, suggesting that the outcome of autophagy modulation is highly context-dependent, varying with cell type, metabolite, and treatment regimen. A notable research gap is the relative paucity of studies examining the stage-specific effects of these metabolites within Correa’s cascade. Most studies focus on established cancer cell lines, with limited investigation into how these agents impact the transition from precancerous lesions to invasive cancer. A classification of these phytochemicals by chemical class and stage of action is provided in Table 2.

**TABLE 2:** Classification of phytochemicals targeting autophagy in Correa’s cascade.

Chemical class	Representative compound	Key autophagy target	Stage(s) of action
Phenolic acids	Chlorogenic acid, syringic acid, ellagic acid, corilagin	Nrf2, AKT/mTOR, LC3-II	Gastritis, cancer
Polyphenols (stilbenes)	Resveratrol, piceatannol, pterostilbene	PI3K/AKT, mTOR, Beclin-1, p62	Precancerous, cancer
Flavonoids	Quercetin, kaempferol, apigenin, baicalein, naringin, diosmetin, hesperidin, nobiletin, isorhamnetin, pectolinarigenin, myricetin, cynaroside, eriodictyol	PI3K/AKT/mTOR, MAPK, AMPK, LC3-II, Beclin-1	All three stages
Alkaloids	Berberine, matrine, magnoflorine, tetrandrine, solanine, harmine	MAPK, PI3K/AKT, mTOR, p62, lysosomal function	Gastritis, cancer
Terpenoids	Astragaloside IV, ginsenoside Rg3/Rg5, patchouli alcohol, isoalantolactone, tanshinone I/IIA, celastrol, β-elemene, oridonin, Jaridon 6	PI3K/AKT/mTOR, HIF-1α, PDCD4-ATG5, p53	Precancerous, cancer
Saponins	Polyphyllin I, A-24, Panax notoginseng saponins, glycyrrhizic acid	PI3K/AKT, HMGB1, lysosome formation	Gastritis, cancer
Organosulfur compounds	Allicin, sulforaphane	PI3K/AKT, p38, Nrf2, miR-4521/PIK3R3	Gastritis, cancer
Carotenoids	Astaxanthin, fucoxanthin	PI3K/AKT/mTOR, AMPK, MMPs	Gastritis, cancer
Lignans	Magnolol	PI3K/AKT, PINK1–PRKN, LC3–OPTN	Cancer
Polysaccharides	Dendrobium officinale polysaccharide, dendrobium kimura polysaccharide	AMPK/mTOR, Wnt/β-catenin	Gastritis, precancerous
Others (mixed)	Cinnamaldehyde, thymoquinone, perillaldehyde, plumbagin, betulinic acid, dihydroartemisinin, decursin, 3,3′-diindolylmethane, proanthocyanidins, Chinese dragon’s blood extract, *Celastrus orbiculatus* extract, *Kaempferia parviflora* (KP-SeNP)	Multiple (PI3K/AKT/mTOR, MAPK, AMPK, NF-κB, ER stress)	All three stages (context-dependent)

## 5 Potential candidates of phytochemicals for the Correa’s cascade of gastric cancer

Because of their multi-step actions on Correa’s cascade and multi-phenotypic regulation, phytochemicals offer advantages over conventional drugs for GC, including multifaceted and long-term effects. Phytochemicals act at multiple stages of Correa’s cascade to halt and potentially reverse gastric carcinogenesis through their diverse pharmacological activities. They exert multiple biological effects, such as anti-inflammatory and anti-*H. pylori* activities, the induction of autophagy and apoptosis, and the modulation of multiple signaling pathways involved in GC development, making them a current research hot spot for the prevention and treatment of GC. In this section, we discuss phytochemicals that play a role in Correa’s cascade.

In the included studies, the experimental doses and treatment durations of berberine, quercetin, and resveratrol that produced significant effects (*p* < 0.05) were combined into a time–dose schedule, as shown in Figure 3. According to the findings, a quercetin concentration of at 25 μM for 48 h was effective during the initial inflammatory prevention phase, whereas concentrations of 20–40 μM for 48 h were effective during the GC inhibition phase. A resveratrol concentration of 50 μM for 48 h was effective during the second GPL reverse phase, whereas concentrations of 50–100 μM for 24–48 h were effective during the third GC inhibition phase. A berberine dose of 15 mg/kg for 23 days effectively reversed GPL progression, whereas concentrations of 4–25 μM for 24–48 h were effective during the GC inhibition phase.

**FIGURE 3:**
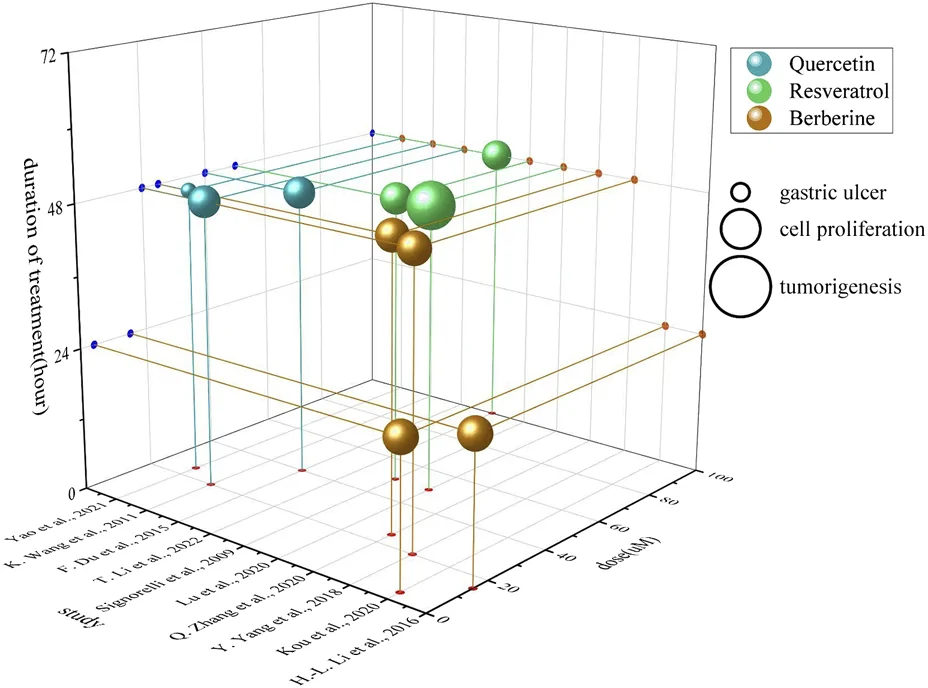
Scatter plot for time–dose interval analysis.

## 6 Future prospects of phytochemicals

Phytochemicals have low toxicity and fewer side effects. As studies on the pharmacological activity of phytochemicals continue, their anti-inflammatory, anti-tumor, and chemotherapeutic-resistant effects have become apparent (Ullah et al., 2025; Nelen et al., 2025). Phytochemicals inhibit the growth and metastasis of various tumor cells at low concentrations, with no significant toxic effects in mouse models. Several studies have reported additive or synergistic effects in certain experimental systems, but these findings require independent replication and rigorous dose-response studies. None of the reviewed studies performed formal synergy analysis in the context of autophagy modulation in gastric cancer. Recent research has found that the combined use of phytochemicals enhances therapeutic efficacy through synergistic mechanisms and that new approaches have been developed to improve the bioavailability of phytochemicals. These synergistic interactions not only potentiate the anti-inflammatory and anti-cancer effects but also modulate autophagy-related pathways in more effectively. The overall scheme of phytochemical actions on autophagy across Correa’s cascade is illustrated in Figure 4.

**FIGURE 4:**
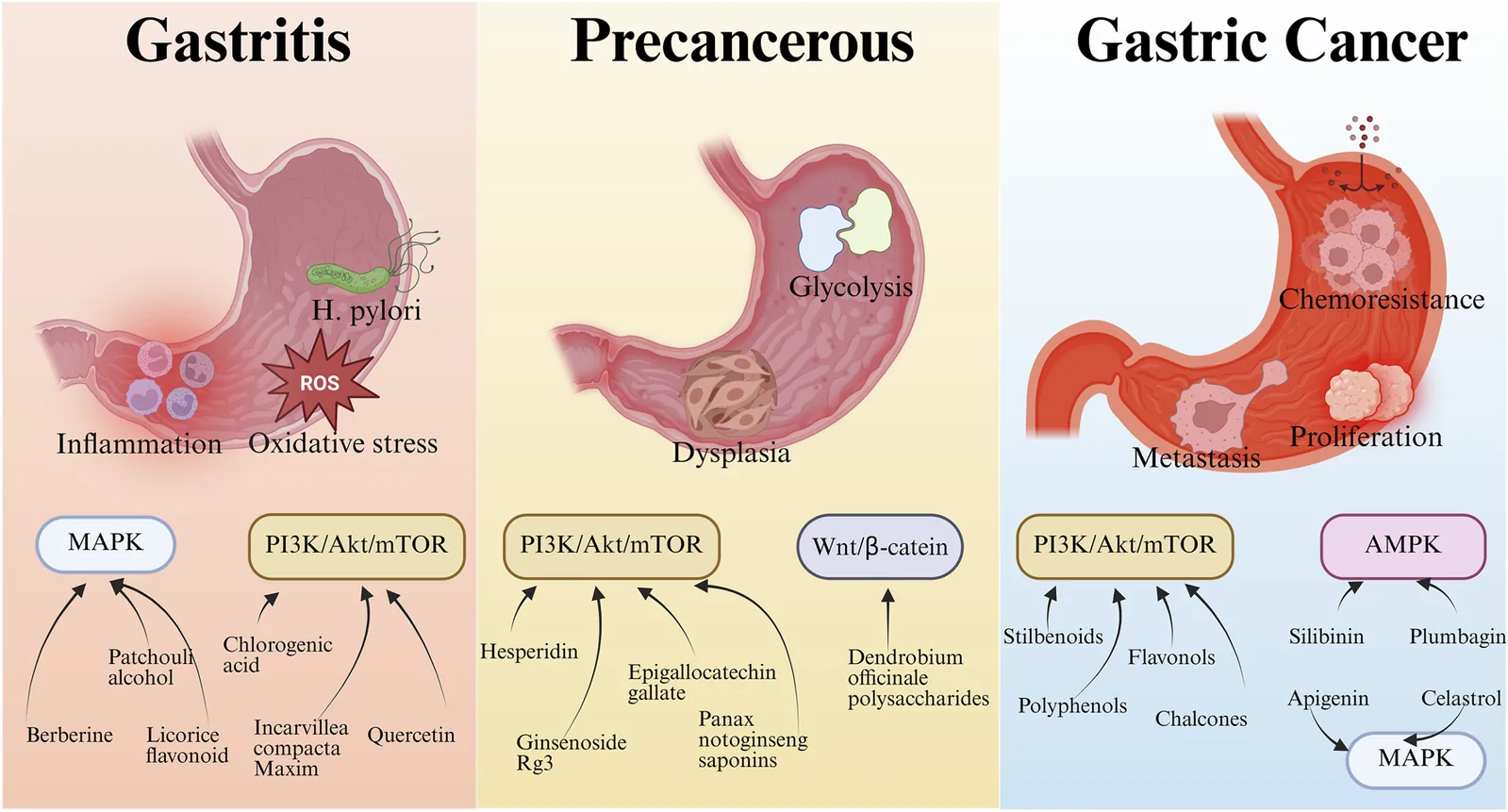
Phytochemicals target the autophagy pathway to inhibit gastric carcinogenesis across the three stages of Correa’s cascade. The left (gastritis) shows metabolites such as berberine, chlorogenic acid, and patchouli alcohol that inhibit MAPK signaling (p38, JNK, and ERK) and modulate PI3K/AKT, thereby reducing inflammation and oxidative stress and promoting mucosal healing. The middle panel (gastric precancerous lesions) features ginsenoside Rg3, astragaloside IV, and Panax notoginseng saponins, which suppress the PI3K/AKT/mTOR axis and Wnt/β-catenin pathway, restoring autophagic flux (increased LC3-II and decreased p62) and reversing epithelial–mesenchymal transition. The right (gastric cancer) illustrates the dual role of autophagy: many metabolites (quercetin, curcumin, and berberine) induce cytoprotective autophagy by inhibiting PI3K/AKT/mTOR, whereas others (tanshinone I, bufalin, and thymoquinone) trigger autophagic cell death through MAPK/ERK or AMPK activation.

For instance, the combination of resveratrol and quercetin has been shown to synergistically activate the AMPKα1/SIRT1 signaling pathway, thereby attenuating high-fat diet-induced circulating inflammatory markers such as TNF-α, IL-6, and monocyte chemoattractant protein-1 (MCP-1) in rats. This synergistic effect may be partly attributed to their combined modulation of autophagy as both metabolites are known to influence the AMPK/mTOR axis (Zhao L. et al., 2017). Piperine (20 mg/kg) with curcumin (100, 200 mg/kg) synergistically lowered brain TNF-α and caspase-3 levels compared to their effects alone in olfactory bulbectomy-induced depression rats (Rinwa et al., 2013). Furthermore, curcumin combined with sulforaphane or phenethyl isothiocyanate synergistically inhibited LPS-induced inflammation in RAW-264.7 cells, which was evidenced by a decrease in inducible nitric oxide synthase (iNOS) and cyclooxygenase-2 (COX-2) protein expression. This combination has also been found to synergistically induce autophagy by promoting LC3-II conversion and inhibiting p62 accumulation, highlighting the potential of multi-targeted phytochemical cocktails. A synergistic interaction between Astragali Radix and Rehmanniae Radix in a traditional Chinese botanical drug formula promotes diabetic wound healing (Lau et al., 2012). Researchers also found that phytochemicals can enhance the bioavailability/uptake of each other. The absorption of rosmarinic acid in Caco-2 cells is significantly enhanced in the presence of luteolin and APG because they inhibit the efflux of rosmarinic acid by inhibiting ABC transporters (Fale et al., 2013).

There are also some new approaches to upgrading the efficacy of phytochemicals. Phytosomes can effectively improve the bioavailability of phytochemicals (Alving, 1991); in a liposomal Polystachya leaf delivery system, hydrogen peroxide scavenging capacity and cellular antioxidant activity were enhanced compared with those of the *Polystachya* leaf extract alone (Wang N. et al., 2024). Phytosome formulations also improved the permeability of curcumin across the endothelial cell monolayer of the blood–brain barrier (BBB) and enhanced its stability against digestive enzymes in brain disease models (Chen and Chiang, 2020). Furthermore, the silybin phytosome improved its lipophilic properties and enhanced its oral bioavailability (Yanyu et al., 2006), while the evodiamine–phospholipid complex increased evodiamine oral bioavailability to 218.82% in rats (Tan et al., 2012). Curcumin phytosomes had better effects of promoting oral absorption and prolonging the retention time of curcumin than single curcumin (Zhang et al., 2013). Nanotechnologies are another way to improve the therapeutic efficacy of phytochemicals. Chitosan nanoparticles can effectively delay EGCG release, enhance its intestinal absorption, and reduce treatment frequency (Dube et al., 2010). In murine models, nanoparticles increased bioavailability, peak blood concentrations, and tumor distribution of curcumin and suppressed tumor/carcinoma growth and angiogenesis. Nano-curcumin, compared to free curcumin, resulted in a 50% reduction in prostate tumor growth (Yallapu et al., 2014). Comparison of nano quercetin and free quercetin at 0–50 μmol/L in breast cancer, apoptosis, and aqueous solubility improved by 1,000-fold (Sun et al., 2014). Although both of these approaches have been reported to enhance the anti-cancer activities of phytochemicals in many *in vitro* and *in vivo* studies, concerns remain regarding their cost, safety, side effects, and long-term toxicity. Currently, the potential toxicity of nanoparticles, encapsulation efficacy, and loading capacity of nanoparticles may cause adverse effects (Desai, 2012).

The studies reviewed herein exhibit considerable heterogeneity in experimental design, which may affect the comparability and generalizability of their findings. First, the majority of *in vitro* studies use established gastric cancer cell lines (AGS, MKN-45, and SGC-7901), which do not fully recapitulate the tumor microenvironment or the genetic and epigenetic diversity of human gastric tumors. Second, animal models vary widely, ranging from acute chemical-induced injury (ethanol/indomethacin) to chronic carcinogen-induced models (MNNG) and xenografts, each with different relevance to human disease. Acute models are useful for studying inflammation but poorly represent the slow, multifactorial progression of Correa’s cascade. Third, the dosage and treatment duration of phytochemicals differ markedly between studies, and many use concentrations far exceeding achievable plasma levels in humans, raising concerns about clinical translatability. Fourth, autophagy assessment is often limited to static markers (LC3-II and p62) without measuring autophagic flux (using lysosomal inhibitors), making it difficult to distinguish between autophagic induction and blockade. Fifth, many of the phytochemicals discussed, including curcumin, resveratrol, quercetin, and several flavonoids, are known pan-assay interference compounds (PAINS). These metabolites can produce false-positive bioactivity readouts through non-specific mechanisms such as redox cycling, aggregation, fluorescence interference, or metal chelation. None of the studies we reviewed performed orthogonal assays to confirm target-specific effects. Therefore, some of the reported autophagy-modulating activities may be artefactual rather than pharmacologically relevant. Finally, no direct head-to-head comparisons between different phytochemicals or between phytochemicals and standard chemotherapy drugs were conducted within a single study, making it impossible to rank their relative efficacy or potency. Finally, few studies compare the effects of the same metabolite across all three stages of the cascade, limiting our understanding of stage-specific actions. Future research should standardize models, include flux assays, and prioritize *in vivo* validation with clinically relevant doses.

Furthermore, the wide variation in experimental models and assay conditions precludes a direct meta-analysis of phytochemical potency; our newly added Table 3 provides a semi-quantitative guide but should be interpreted with caution. Future studies should adopt more standardized protocols to enable meaningful quantitative comparisons across metabolites. Based on these limitations, we propose the following priorities for future research: a. mandatory inclusion of autophagic flux assays and orthogonal PAINS validation, b. use of clinically relevant concentrations, c. more realistic chronic disease models (*H. pylori*-infected transgenic mice), d. head-to-head comparative studies, e. pilot human trials with gastric biopsy-based autophagy readouts, and f. rational combination strategies with autophagy inhibitors.

**TABLE 3:** Quantitative comparison of representative phytochemicals in gastric cancer models.

Phytochemical	Model system	Effective concentration/dose	Autophagy-related readouts	Key outcome
Quercetin	AGS	20–40 μM (48 h)	LC3-II ↑, Beclin-1 ↑, p62 ↓, p-AKT ↓, p-mTOR ↓	Protective autophagy; co-treatment with CQ/3-MA increases apoptosis
Quercetin	H_2_O_2_-induced GES-1	25–50 μM	PI3K/AKT activation (protective), ROS ↓, MMP ↓	Attenuates oxidative stress-induced damage
Resveratrol	SGC-7901	50–100 μM (24–48 h)	LC3-II ↑, p62 ↑, Beclin-1 ↓, p-AKT ↓, p-mTOR ↓, p38 MAPK ↑, ERK ↑	Induces ER stress and protective autophagy; enhances chemosensitivity
Resveratrol	HGC-27	50 μM (24 h)	LC3-II ↑ (*via* dihydroceramide accumulation), autophagic flux ↑	Promotes autophagy through the ceramide pathway
Resveratrol	GES-1	50 μM (48 h)	PI3K/AKT ↓, p-FoxO4 ↑, CDX2 ↓	Reverses intestinal metaplasia (precancerous)
Curcumin	BGC-823, SGC-7901	15–30 μM (24–48 h)	AVOs ↑, LC3-II ↑, ATG5 ↑, ATG7 ↑, p-PI3K ↓, p-AKT ↓, p-mTOR ↓	Protective autophagy; autophagy inhibition enhances apoptosis
Berberine	BGC-823	25 μM (48 h)	AVOs ↑, LC3-II ↑, Beclin-1 ↑, p62 ↓, MAPK/mTOR/p70S6K ↓	Cytostatic autophagy enhances cisplatin sensitivity
Berberine	MGC-803	4–25 μM (24–48 h)	p-p38 ↓, p-ERK1/2 ↓, p-JNK ↓, IL-8 ↓	Anti-inflammatory, inhibits proliferation
Berberine	Atp4a−/− mice	15 mg/kg (23 days)	PI3K/AKT/mTOR ↓, LDHA ↓, HIF-1α ↓, ROS ↑	Reverses gastric precancerous lesions
Ginsenoside Rg3	Atp4a−/− mice	20 mg/kg (12 weeks)	PI3K/AKT/mTOR ↓, LDHA ↓, HK-II ↓, HIF-1α ↓	Inhibits glycolysis, delays PLGC progression
Astragaloside IV	MNNG-induced rat PLGC	80 mg/kg (12 weeks)	p53 ↑, miRNA-34a ↑, LDHA ↓ (glycolysis), Beclin-1 ↓, LC3-II ↓	Reverses precancerous lesions *via* p53/miRNA-34a axis
Astragaloside IV	BGC-823, MKN-74	50–200 μM (48 h)	PI3K/AKT/NF-κB ↓, E-cadherin ↑, N-cadherin ↓, vimentin ↓	Inhibits EMT and metastasis
Chlorogenic acid	Indomethacin-induced gastric ulcer (rat)	40 mg/kg (oral)	LC3-II/LC3-I ↓, Beclin-1 ↓, p62 ↓, AKT/mTOR ↑ (normalization)	Restores autophagy to basal levels, protects gastric mucosa
Chlorogenic acid	Dexamethasone-stressed broiler gut	400 mg/kg diet	Nrf2 ↑, Beclin-1 ↑, ATG5 ↑, ATG7 ↑, LC3-II ↑	Induces protective autophagy *via* Nrf2
Tanshinone I	BGC-823, SGC-7901	5 μM (24 h)	LC3-II ↑, Bcl-2 ↓, Beclin-1-Bcl-2 complex ↓, cleaved caspase-3 ↑	Cytoprotective autophagy; combination with CQ increases apoptosis
Bufalin	MGC-803	20–100 nM (24–48 h)	LC3-II ↑, Beclin-1 ↑, p62 ↓, p-AKT/mTOR/p70S6K ↓, ERK ↑	Induces ER stress-dependent cytoprotective autophagy
Thymoquinone	HGC-27, MKN-45	10–50 μM (24–48 h)	LC3-II ↑, p62 ↓, p-PI3K ↓, p-AKT ↓, p-mTOR ↓	Protective autophagy; combination with BafA1 enhances apoptosis
Kaempferol	AGS, SNU-216	25–100 μM (24 h)	LC3-II ↑, AMPK/ULK1 ↑, IRE1-JNK-CHOP pathway ↑, G9a ↓	Autophagic cell death (non-protective)
Apigenin	AGS	20–80 μM (24–48 h)	LC3-II ↑, Beclin-1 ↑, p62 ↓, p-PI3K ↓, p-AKT ↓, p-mTOR ↓	Protective autophagy (blocked by 3-MA)
Naringin	AGS	200–800 μM (24 h)	AVOs ↑, LC3-II ↑, Beclin-1 ↑, p-ERK1/2 ↑, p-p38 ↑, p-JNK ↑, PI3K/AKT/mTOR ↓	Induces protective autophagy *via* MAPK activation
Eugenol	MNNG-induced rat GC model	100 mg/kg (20 weeks)	NF-κB ↓, p53 ↑, p21 ↑, LC3-II ↑, p62 ↑	Induces autophagy and suppresses carcinogenesis
Allicin	SGC-7901	10–40 μg/mL (24–48 h)	PI3K/AKT ↓, p38 ↓, ROS ↑, G2/M arrest	Induces apoptosis and protective autophagy
Dihydroartemisinin	SGC-7901	25–100 μM (24–48 h)	LC3-II ↑, p62 ↓, p-AKT ↓, E-cadherin ↑, Snail ↓	Reverses EMT, induces protective autophagy
Pterostilbene + sorafenib	N-87, MKN-45	50 μM pterostilbene +2.5 μM sorafenib (48 h)	AVOs ↑, LC3-II ↑, p62 ↓, autophagic flux ↑	Synergistic anti-tumor effect *via* autophagy
Corilagin	SGC-7901, BGC-823	20–80 μM (24–48 h)	LC3-II ↑, AVOs ↑, ROS ↑, p-PI3K ↓, p-AKT ↓, Bcl-2 ↓	Induces both apoptosis and autophagy

IC_50_ values are reported as approximate ranges from the cited studies; exact values may vary with cell line and experimental conditions.

AVOs, acidic vesicular organelles; LC3-II/I ratio or LC3-II, levels indicate autophagosome formation; p62/SQSTM1 degradation indicates autophagic flux; CQ, chloroquine; 3-MA, 3-methyladenine; BafA1, bafilomycin A1; GPL, gastric precancerous lesion; PLGC, precancerous lesions of gastric cancer; IM, intestinal metaplasia.

## 7 Conclusion

The development of GC is a continuous process of pathological changes, as described by Correa’s cascade. This review systematically maps the landscape of phytochemicals that target autophagy across the three distinct stages of this cascade, moving beyond a simple catalog of metabolites to an analysis of their mechanistic convergence on key signaling hubs, predominantly the PI3K/AKT/mTOR and MAPK pathways. A key finding is the stage-dependent duality of autophagy: protective during inflammation, tumor-suppressive in precancerous lesions, and frequently cytoprotective in established cancer. This nuanced understanding is crucial for the development of stage-specific therapeutic strategies.

A significant gap identified is the relative scarcity of research on phytochemical-mediated autophagy during the gastritis and precancerous stages, which represent the most critical windows for intervention and reversal of the disease process. Furthermore, although the concept of synergy has been proposed, rigorous studies investigating the combined effects of phytochemicals on the simultaneous modulation of multiple autophagy-related pathways remain lacking. Future research should prioritize: 1. elucidating the molecular mechanisms of phytochemicals in the early stages of Correa’s cascade. 2. Investigating the synergistic potential of phytochemical combinations to overcome cytoprotective autophagy in GC. 3. Leveraging advanced formulations such as phytosomes and nanoparticles to enhance bioavailability and clinical efficacy. By addressing these gaps, phytochemicals that precisely modulate autophagy hold immense promise as next-generation, low-toxicity agents for the prevention and treatment of gastric cancer.
