# Supplementary material for: Targeting the Correa’s cascade of gastric cancer: the potential role of phytochemicals and autophagy modulation
Source: Front Pharmacol. 2026 Sep 25;17:1689279. doi: 10.3389/fphar.2026.1689279 (PMC13650431; doi:10.3389/fphar.2026.1689279)
Supplement: Supplementary file 3 [file Supplementaryfile1.pdf]

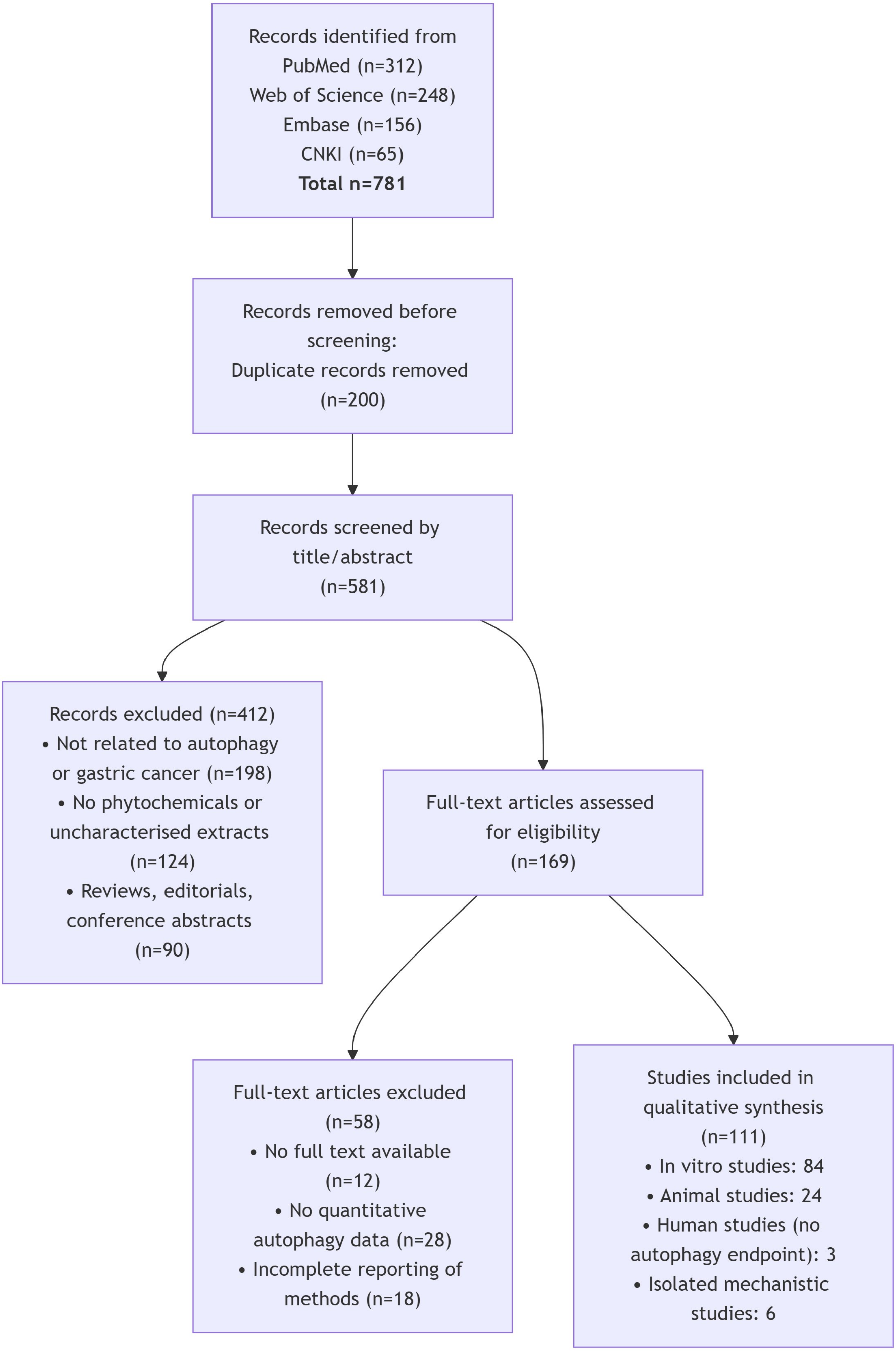
Records identified from
PubMed (n=312)
Web of Science (n=248)
Embase (n=156)
CNKI (n=65)
Total n=781
Records removed before screening:
Duplicate records removed (n=200)
Records screened by title/abstract (n=581)
Records excluded (n=412)
• Not related to autophagy or gastric cancer (n=198)
• No phytochemicals or uncharacterised extracts (n=124)
• Reviews, editorials, conference abstracts (n=90)
Full-text articles assessed for eligibility (n=169)
Full-text articles excluded (n=58)
• No full text available (n=12)
• No quantitative autophagy data (n=28)
• Incomplete reporting of methods (n=18)
Studies included in qualitative synthesis (n=111)
• In vitro studies: 84
• Animal studies: 24
• Human studies (no autophagy endpoint): 3
• Isolated mechanistic studies: 6
